# A reverse genetics system for avian coronavirus infectious bronchitis virus based on targeted RNA recombination

**DOI:** 10.1186/s12985-017-0775-8

**Published:** 2017-06-12

**Authors:** Steven J. van Beurden, Alinda J. Berends, Annika Krämer-Kühl, Dieuwertje Spekreijse, Gilles Chénard, Hans-Christian Philipp, Egbert Mundt, Peter J. M. Rottier, M. Hélène Verheije

**Affiliations:** 1 Faculty of Veterinary Medicine, Department of Pathobiology, Utrecht University, Yalelaan 1, 3584 CL Utrecht, The Netherlands; 2 Boehringer Ingelheim Veterinary Research Center GmbH & Co. KG, Bemeroder Str. 31, 30559 Hannover, Germany; 3 Boehringer Ingelheim Animal Health Operations, C.J. van Houtenlaan 36, 1381 CP Weesp, The Netherlands

**Keywords:** Avian coronavirus, Infectious bronchitis virus, Mouse hepatitis virus, Targeted RNA recombination, Reverse genetics system, Vaccine development, Chicken, Poultry, Embryonated eggs

## Abstract

**Background:**

Avian coronavirus infectious bronchitis virus (IBV) is a respiratory pathogen of chickens that causes severe economic losses in the poultry industry worldwide. Major advances in the study of the molecular biology of IBV have resulted from the development of reverse genetics systems for the highly attenuated, cell culture-adapted, IBV strain Beaudette. However, most IBV strains, amongst them virulent field isolates, can only be propagated in embryonated chicken eggs, and not in continuous cell lines.

**Methods:**

We established a reverse genetics system for the IBV strain H52, based on targeted RNA recombination in a two-step process. First, a genomic and a chimeric synthetic, modified IBV RNA were co-transfected into non-susceptible cells to generate a recombinant chimeric murinized (m) IBV intermediate (mIBV). Herein, the genomic part coding for the spike glycoprotein ectodomain was replaced by that of the coronavirus mouse hepatitis virus (MHV), allowing for the selection and propagation of recombinant mIBV in murine cells. In the second step, mIBV was used as the recipient. To this end a recombination with synthetic RNA comprising the 3′-end of the IBV genome was performed by introducing the complete IBV spike gene, allowing for the rescue and selection of candidate recombinants in embryonated chicken eggs.

**Results:**

Targeted RNA recombination allowed for the modification of the 3′-end of the IBV genome, encoding all structural and accessory genes. A wild-type recombinant IBV was constructed, containing several synonymous marker mutations. The *in ovo* growth kinetics and in vivo characteristics of the recombinant virus were similar to those of the parental IBV strain H52.

**Conclusions:**

Targeted RNA recombination allows for the generation of recombinant IBV strains that are not able to infect and propagate in continuous cell lines. The ability to introduce specific mutations holds promise for the development of rationally designed live-attenuated IBV vaccines and for studies into the biology of IBV in general.

**Electronic supplementary material:**

The online version of this article (doi:10.1186/s12985-017-0775-8) contains supplementary material, which is available to authorized users.

## Background

*Avian coronavirus* infectious bronchitis virus (IBV) primarily infects the upper respiratory epithelium of chickens, causing a respiratory disease that is frequently complicated by secondary bacterial pathogens [1]. In addition, some IBV strains affect the renal tubuli, oviduct and parts of the gastrointestinal tract, leading to pathological lesions in these organ systems, with subsequent reduced weight gain and a drop in egg production. The virus has a worldwide presence in both commercial and backyard chickens, appearing in a wide variety of geno-, sero- and protectotypes [2]. IBV is currently regarded as one of the economically most relevant viral pathogens in the poultry industry.

**Fig. 1:**
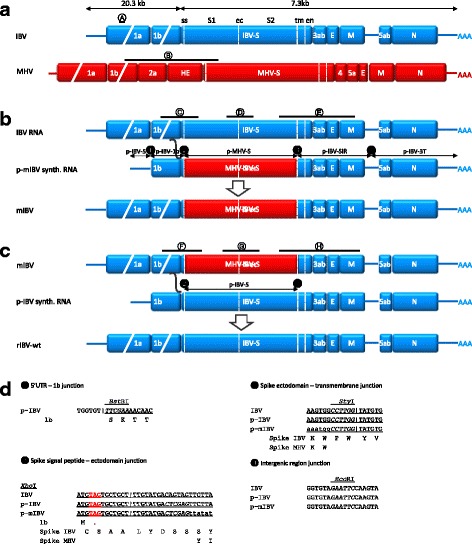
Coronavirus genome organization and schematic overview of targeted RNA recombination. **a** Schematic genome representations of IBV (*blue*) and MHV (*red*). The first two-third of the genome is truncated, the structural and accessory genes are drawn to scale. The lengths of the first two-thirds and last one-third of the IBV genome are indicated at the top. The different domains of the spike gene are indicated: ss = signal sequence; ec = ectodomain; tm = transmembrane domain; en = endodomain. PCR amplicons are depicted as black bars drawn to scale above the genomes, with encircled letters referring to the primer sets in Table 3 and Fig. 3. **b** Stage 1 in targeted RNA recombination: an interspecies chimeric murinized IBV with a MHV spike ectodomain (mIBV) is generated by a single recombination event of IBV genomic RNA with synthetic RNA transcribed from donor plasmid p-mIBV in the 3′-end region of the 1b gene (indicated by a *black* curved line). Murinized IBV is selected on murine LR7 cells. Plasmid inserts are indicated above p-mIBV, with numbers in black circles referring to the plasmid junctions. **c** Stage 2 in targeted RNA recombination: a recombinant IBV with the IBV spike gene (rIBV) is recreated by a single recombination event of mIBV with synthetic RNA transcribed from donor plasmid p-IBV. Recombinant IBV is selected on embryonated chicken eggs. **d** Nucleotide sequences of the plasmid junctions, marked with corresponding numbers in the schematic donor plasmid drawings. Nucleotide sequences are indicated for wild-type IBV and donor plasmids p-IBV and p-mIBV, with restriction enzyme sites in italics, MHV spike gene sequences in lower case, and spike domains (i.e. ss, ec, tm and en) separated by vertical dashes. Stop codons are highlighted in red. Open reading frames (ORFs) are underlined, overlapping ORFs are double-underlined, and ORF translations are indicated as amino acids below the nucleotide sequences if applicable

Infectious bronchitis virus is the prototype gammacoronavirus in the family *Coronaviridae*, order *Nidovirales* [3]*.* The enveloped virus particles have a positive-sense RNA genome of 27.6 kb (Fig. 1a) [4]. The 5′ two-third of the viral genome comprises gene 1, divided into two large open reading frames 1a and 1b, which code for 15 nonstructural proteins primarily involved in RNA replication and transcription. The 3′ one-third of the viral genome codes for structural proteins: spike protein (S, encoded by gene 2), envelope protein (E, encoded by gene 3c), and membrane protein (M, encoded by gene 4), each located in the viral envelope. The nucleocapsid protein (N, encoded by gene 6) occurs in the ribonucleoprotein core [5]. Interspersed between the structural genes, coronaviruses carry a variable number of genus specific accessory genes [6]. Most of their gene products are nonstructural, and their expression is not essential for virus replication in vitro [7–12]. The IBV genome contains the accessory genes 3 and 5, encoding the proteins 3a and 3b, and proteins 5a and 5b, respectively [4]. In addition, an open reading frame located in the intergenic region was identified between genes 4 and 5 [13].

The typical coronavirus spikes are formed by trimers of the type 1 membrane protein S, which is often proteolytically cleaved into two subunits, S1 and S2 [4, 14]. The glycosylated S1 domain forms the ‘head’ of the spike and contains the receptor-binding domain [15]. Avian gammacoronaviruses typically interact with glycans on the host cell surface. IBV in particular requires α2,3-linked sialic acids for attachment and entry [16–18]. The S2 domain builds the remaining part of the ectodomain (the ‘stalk’), the transmembrane domain and the internally located endodomain. The S protein is the main determinant of the coronaviral host species tropism [19].

Many mammalian coronaviruses of the genera *Alphacoronavirus* and *Betacoronaviruses* can be propagated in cultured cells, unlike most avian coronaviruses of the genus *Gammacoronavirus*. IBV can, however, readily be propagated in, and isolated from, embryonated fowl eggs. During passaging in embryonated eggs adaptation occurs, often leading to attenuation. For example, IBV strain H52 represents the 52nd serial passage of a Massachusetts-like IBV strain isolated in The Netherlands [20], which causes embryonic death within 48 h post infection (hpi), and still has a residual virulence in young chickens. Another 68 passages resulted in the IBV strain H120, which is more attenuated and has a lower pathogenicity in young chicks. Similar serial passaging of another IBV strain of the Massachusetts serotype isolated in the USA resulted in the generation of the non-pathogenic and cell-culture adapted IBV strain Beaudette [21].

In order to study its characteristics, several research groups have independently developed a reverse genetics system (RGS) for IBV which allow the manipulation of its genome [22–26]. All these systems are based on the non-pathogenic cell-culture adapted IBV strain Beaudette, or the highly attenuated IBV vaccine strain H120. A major drawback of the use of the non-virulent IBV strains Beaudette and H120 [20, 21] is the inability to provide insights in the infection process in chickens, as these strains no longer cause a clinically relevant phenotype in vivo. Yet, the RGS has provided significant insight in the fundamentals of avian gammacoronavirus replication. Key findings include that IBV cell tropism is determined by the spike gene [27, 28], that the low virulence of IBV Beaudette is caused by changes in the replicase gene [29], and that one or more of the IBV accessory gene products interfere with the hosts’ interferon response [30–32].

Targeted RNA recombination is another reverse genetics approach, so far only developed for mammalian coronaviruses from the genera *Alphacoronavirus* and *Betacoronavirus* [11, 19, 33]. This system is based on the exchange of the spike gene by that of a coronavirus with a different host tropism, which enables subsequent selection on cells susceptible to the heterologous species [34]. As a consequence, manipulation is limited to the last third of the coronavirus genome, covering all genes encoded that are located 3′ of gene 1, starting with the spike gene. Targeted RNA recombination has been shown to be easy in use and to allow the rescue of highly defective mutants [11, 19, 33]. However, the system is based on the ability to propagate both the donor and the recipient coronavirus in cell culture, and is hence not implementable for pathogenic IBV. This problem was solved by transfecting IBV genomic RNA into otherwise non-susceptible cells, exchanging the IBV spike gene by that of the mouse hepatitis virus (MHV) provided as part of a synthetic RNA, and by subsequently rescuing recombinant IBV from infected/transfected cells in embryonated eggs (Fig. 1). This system was successfully established to introduce marker mutations in the last one-third of the genome of IBV. The resulting recombinant viruses demonstrated growth kinetics *in ovo* and the in vivo phenotypic characteristics in one-day-old chickens similar to IBV wild type. The results presented here demonstrate for the first time a host species switch for an avian gammacoronavirus by exchanging the spike gene with that of the highly divergent betacoronavirus MHV. This RGS enables the manipulation of the structural and accessory protein genes from the genome of virulent IBV.

## Methods

### Cells, eggs, viruses & antibodies

Baby hamster kidney (BHK-21) cells (ATCC CCL-10) and murine LR7 cells KUO2000 were cultured in Dulbecco’s Modified Eagle Medium (DMEM) (BioWhittaker), supplemented with 4 mM L-glutamine (Lonza, Basel, Switzerland), 10% fetal bovine serum (FBS) (BioWhittaker) and 0.05 mg/ml gentamicin (Gibco Invitrogen), at 37 °C and 5% CO_2_. Virus titers in cells were obtained by determining the 50% tissue culture infective dose (TCID_50_) per ml at 2 days post inoculation (p.i.) according to the Spearman–Kärber method [35].

Fertilized specific pathogen free (SPF) white leghorn eggs (Animal Health Service, Deventer, The Netherlands) were incubated at 37.5 °C and 45–65% relative humidity. Eight-day- embryonated chicken eggs (ECE) were inoculated via the allantoic cavity unless stated otherwise, and candled twice daily. Upon embryonic death or no later than 7 days p.i., eggs were transferred to 4 °C for 16–24 h prior to allantoic fluid (AF) and chorio-allantoic membrane (CAM) collection. Virus titration *in ovo* was based on the determination of the 50% embryonic infectious dose (EID_50_) per ml, as determined at day 7 p.i. according to Reed and Muench [36]. For the production of a virus stock, ten 8-day-old ECE were inoculated with 100 EID_50_, incubated for 24 h, and subsequently cooled for 16–24 h before the AF was harvested and pooled.

IBV strain H52 (Boehringer Ingelheim (BI), Ingelheim, Germany) was propagated in embryonated SPF eggs and titrated. IBV strain Beaudette (Animal Health Service, Deventer, The Netherlands) was propagated and titrated in BHK-21 cells. Mouse hepatitis virus (MHV) strain A59 was propagated and titrated in LR7 cells.

Monoclonal antibody (MAb) Ch/IBV 26.1 against the IBV S2 protein was obtained from Prionics (Thermo Fisher Scientific, Waltham, MA, USA) [37, 38]. The production of rabbit polyclonal antiserum k134 against MHV was described previously [39]. Chicken polyclonal antiserum was derived from a SPF chicken vaccinated with IBV strain H120 (BI, Ingelheim, Germany). Secondary fluorescently-labeled antibodies Alexa Fluor 488 goat anti-chicken IgY, Alexa Fluor 568 goat anti-rabbit IgG, and Alexa Fluor 488 goat anti-mouse IgG (Invitrogen by Thermo Fisher Scientific) were stored in 50% glycerol at −20 °C.

### Anti-IBV immuno histochemistry

CAMs were collected from ECEs, washed in PBS, fixed in neutral buffered 10% formalin in PBS for 24 h, stored in 70% ethanol and finally paraffin-embedded. Four micrometer sections of CAM were mounted on glass slides and subsequently deparaffinized and rehydrated in alcohol series. Next, the sections were subjected to endogenous peroxidase inactivation and antigen retrieval as described before [40]. Sections were washed in phosphate buffered Normal Antibody Diluent (NAD, ScyTek Laboratories, Logan, USA) containing 0.1% Tween-20, and after primary antibody incubation with PBS 0.1% Tween-20. Sections were incubated for 60 min at room temperature with MAb Ch/IBV 26.1 diluted 1:100 in NAD. Antibody binding was detected by Dako Envision HRPO labeled polymer anti-mouse (Dako, by Agilent Technologies, Santa Clara, USA) diluted 1:1 in NAD, and visualized by 3-Amino-9-ethylcarbazole (AEC, Dako). Slides were counterstained with hematoxylin, mounted with Aquatex (Merck, Darmstadt, Germany), and viral antigen presence was assessed by light microscopy (BX60, Olympus, Tokyo, Japan).

### RNA isolation, reverse transcription, PCR and RT-qPCR

RNA was isolated from harvested AF using the QIAamp viral RNA Mini Kit (Qiagen, Hilden, Germany) according to manufacturer’s protocol. Reverse transcription (RT) was performed using the Transcriptor First Strand cDNA Synthesis Kit (Roche, Basel, Switzerland) according to manufacturer’s protocol, with random hexamers for standard PCR, or with specific primers for sequencing and cloning purposes. PCR was performed with recombinant Taq DNA polymerase (Thermo Fisher Scientific) for plasmid characterization or with Phusion Hot Start II High-Fidelity DNA Polymerase (Thermo Fisher Scientific) for sequencing and cloning purposes.

One-step RT-qPCR was used to semi-quantitatively assess virus load in AF. Forward primer IBV.RdRp.F41 (3′-CATGCAGTTTGTTGGAGATCCT-5′) and reverse primer IBV.RdRp.R41 (3′-GTGACCTGGTTTTACCGTTTGA-5′) targeting the conserved region of gene 1b (nucleotide position 13,412 to 13,580 in IBV Beaudette GenBank accession number M95169.1) coding for the RNA-dependent RNA polymerase protein. Primers were obtained from Biolegio (Nijmegen, The Netherlands) and used at a final concentration of 300 nM each with the iTaq universal SYBR Green one-step kit (Bio-Rad Laboratories, Hercules, California, USA). The RT-qPCR reaction was carried out in a Bio-Rad CFX Connect real-time PCR system, starting with 10 min at 50 °C and 1 min at 95 °C, followed by 40 cycles of 10 s at 95 °C and 30 s at 60 °C, and ending with a dissociation step for the determination of the melting point of the obtained PCR fragment.

### Construction of IBV donor plasmid

The complete genome sequence of IBV H52 BI was determined by Sanger sequencing using primers as described by Zhou et al. [25]. The 5′- and 3′-UTR sequences were identified using the 2nd generation 5′/3′ RACE kit (Roche, Basel, Switzerland). The IBV H52 BI genome sequence was 27,640 nucleotides (nt) in length, including an annotated 10 nt polyA tail.

The design of the donor plasmids principally followed the strategy previously described by Kuo et al. [19]. The final donor plasmid p-IBV was constructed from the stepwise ligation of fragments derived from five plasmids (Fig. 1b and Table 1, and described below in detail): Plasmid (p)IBV-5 comprises a T7 RNA polymerase promotor, 2 G nucleotides, and the near full-length 5′-untranslated region (UTR), with an unintended A to C substitution at position 54. Plasmid IBV-1b comprises the last 754 nt of the pol 1b gene, including the 50 nt overlap with the spike gene, and the first 66 nt of the spike gene, including the signal sequence. Plasmid IBV-S contains the near full length ectodomain of the spike gene, 3211 nt in length. Plasmid IBV-SIR comprises the last 212 nt of the spike gene (the transmembrane and the endodomain), the accessory gene 3, the envelope gene, the membrane gene and half of the intergenic region. Plasmid IBV-3 T comprises the 3′-terminal region of the IBV genome, including the second half of the IR, the accessory gene 5, the nucleocapsid gene, the 3′-UTR and a 100 nt poly-A sequence. All plasmids were generated by GenScript (Piscataway, NJ, USA) and provided in the plasmid pUC57-simple, a standard cloning plasmid with the polylinker removed.

**Table 1:** Plasmids used for generation of the donor plasmids p-IBV and p-mIBV

Plasmid	Genes	Coordinates	Length (nt)	3′-end RES	Surrounding RES	Inserted at 3′-end of
p-IBV-5	T7	n.a.	18	n.a.	n.a.	n.a.
	GG	n.a.	2	n.a.	n.a.	n.a.
	IBV 5′-UTR	1–497	497	*Bst*BI	n.a.	n.a.
p-IBV-1b	IBV 3′-end 1b, S (ss)	19,610–20,379	770	*Xho*I	*Bst*BI	p-IBV-5
p-IBV-S	IBV S (ec)	20,379–23,590	3211	*Sty*I	*Xho*I	p-IBV-5-1b
p-IBV-SIR	IBV S (tm, en), 3a, 3b, E, M	23,591–25,318	1728	*Eco*RI	*Sty*I	p-IBV-5-1b-S
p-IBV-3 T	IBV 5a, 5b, N, 3′-UTR	25,319–27,630	2322	n.a.	*Eco*RI	p-IBV-5-1b-S-SIR
	PolyA tail	27,631–27,730	100	*Mss*I, *Pac*I	n.a.	n.a.
p-MHV-S	MHV S (ec)	n.a.	3757	*Sty*I	*Xho*I	p-IBV-5-1b

IBV gene fragments were generated in pUC57-simple, the MHV spike ectodomain derived from pTUG was ligated into pJet1.2. Plasmid names and spike protein domain abbreviations refer to Fig. 1. (ss) = signal sequence; (ec) = ectodomain; (tm) = transmembrane domain; (en) = endodomain; n.a. = not applicable. RES = restriction enzyme site

In order to allow cloning of the fragments in a stepwise approach, naturally occurring restriction enzyme sites (RES) located in the viral cDNA were used, except for the *Bst*BI site between p-IBV-5 and p-IBV-1b, which is only partly present in the 5′-UTR, and the *Xho*I site between p-IBV-1b and p-IBV-S, which was introduced without changing the amino acid sequence (silent mutation). Restriction enzyme sites were made unique by silently removing these RES from other parts of the genome included in the donor plasmid (Additional file 1: Table S1). In addition, semi-unique RES were introduced by silent mutations within 200 nt up- and downstream of the accessory genes 3 and 5. Finally, unique RES *Mss*I and *Pac*I were included after the poly-A sequence, allowing linearization of the plasmid by a single restriction enzyme digest. All genome fragments were ligated step-by-step into p-IBV-5 using the restriction enzymes specified in Table 1. Each ligation mixture was subsequently transfected into HB101 competent cells and plasmid DNA was isolated by performing midiprep DNA isolation (Qiagen, Hilden, Germany). The final plasmid consisted of p-IBV-5-1b-S-SIR-3 T, now called p-IBV (Fig. 1c). The composition of each plasmid was confirmed after each cloning step by PCR, restriction enzyme digestion and sequencing of each of the inserts (Macrogen, Amsterdam, The Netherlands).

### Construction of mIBV donor plasmid

The ectodomain of the MHV A59 spike gene was amplified from pTUG [41] by PCR using primers with an *Xho*I overhang (Table 2) and ligated into pJet1.2 resulting in p-MHV-S. Site directed mutagenesis (SDM) with the Q5 SDM kit (New England Biolabs, Ipswich, USA) was used to silently remove an *Eco*RI and an *Xho*I RES interfering with subsequent cloning steps (Table 2). The ectodomain of MHV spike was ligated into p-IBV-5-1b, followed by subsequent cloning steps using the IBV fragments SIR and 3 T. This resulted in the plasmid p-IBV-5-1b-mhvS-SIR-3 T, now called p-mIBV (Fig. 1b).

**Table 2:** Primers used for SDM and cloning of the MHV spike and IBV nucleocapsid gene

Primer	Sequence (5′ ➔ 3′)	Function
XhoI-t-MHV-S.F01	*CTCGAG* **T**TATATTGGTGATTTTAGATGTATCCAG	Cloning MHV S
MHV-S-StyI-XhoI.R01	*CTCGAG*CCAAGGCCATTTCACATACATTTC	Cloning MHV S
p-MHV-S-SDM414.F02	AGCTTGTGAACTCAAACGGTG	SDM *Eco*RI
p-MHV-S-SDM414.R02	GGATACATCTAAAATCACCAATATAAC	SDM EcoRI
p-MHV-S-SDM2823.F03	TTACTATAAGTTCGAGACTGCC	SDM XhoI
p-MHV-S-SDM2823.R03	CACCCTGCATTAATGCAC	SDM XhoI
IBV-H52_N_ATG_FW	ACCATGGCGAGCGGTAAGA	N-transcript
IBV-M41-#2-IR-RV	TTTTTTTTTTTTTTTTTTTGCTCTAACTCTATACTAG	N-transcript

*Xho*I restriction enzyme sites in sequences of primers *Xho*I-t-MHV-S.F01 and MHV-S-*Sty*I-*Xho*I.R01 are in italics; an additional thymidine residue to keep the MHV spike gene ectodomain sequence in frame with the IBV spike gene signal sequence is in bold. SDM = site directed mutagenesis

### Construction of N transcript plasmid

A plasmid comprising the nucleocapsid gene and 3′-UTR sequence of IBV H52 BI was generated by PCR amplifying the respective region using primers IBV-H52.N.ATG.FW and IBV-M41#2.IR.RV (Table 2). The amplicon was ligated into pJet1.2 downstream of the T7 promotor sequence, resulting in p-IBV-N, and the correctness of the insert was verified by sequencing.

### In vitro transcription

Capped, run-off donor transcripts were synthesized from p-IBV, p-mIBV and p-IBV-N using the mMessage mMachine T7 kit (Ambion by Thermo Fisher Scientific). In brief, p-mIBV was *Pac*I-linearized, and p-IBV and p-IBV-N were *Mss*I-linearized. Linearized plasmid DNA was ethanol precipitated. Transcription reactions were prepared according to the manufacturer’s instructions, using 1.5 and 0.5 μg linearized DNA per 10 ul reaction for p-(m)IBV and p-IBV-N, respectively. After 1 h of incubation at 37 °C, production of RNA was verified by analyzing 1 μl of the reaction volume by gel electrophoreses. After an incubation of 2 h the reaction was stopped by transferring the reaction tubes to ice.

### Targeted RNA recombination and rescue of mIBV

The IBV spike gene was replaced by a chimeric MHV-IBV spike gene in the IBV genome by targeted RNA recombination between p-mIBV generated donor RNA and recipient virus (IBV) RNA, as described before [19]. IBV H52 viral RNA was transfected into BHK-21 cells, a cell line known to support replication of IBV [25], but not infection with IBV H52 (data not shown). Thus, 20 μl of IBV H52 BI RNA obtained from allantoic fluid, mixed with 10 μl transcript reaction mixtures of p-mIBV and p-IBV-N each were transfected into BHK-21 cells by electroporation using two pulses at 850 V and 25 μF in a Gene Pulser electroporation apparatus (Bio-Rad). Transfected BHK-21 cells were seeded onto monolayers of LR7 cells having an approximate confluence of 70–80% and incubated at 37 °C.

Two days after seeding, when syncytia in the LR7 monolayer were observed, the cell culture supernatant was harvested and rescued viruses were purified by two rounds of plaque purification on LR7 cells. Characterization of the last one-third of the genome of candidate recombinants was performed by RT-PCR and subsequent Sanger sequencing of the obtained cDNA-fragments, using the primer sets specified in Table 3. Murinized IBV (mIBV) strain #1B3-IIA was selected based on sequence analysis, and virus stocks were propagated and stored at −80 °C. Aliquots were titrated on LR7 cells.

**Table 3:** Primer sets used for genetic characterization of mIBV and rIBV-wt

Primer set	Target	Primer	Sequence (5′ ➔ 3′)	Amplicon (bp)
A	IBV 1a – IBV 1a	IBV.F02	GGTGTAACACCAGAGATAAATG	1416
		IBV.R02	ATTTACGACGTCAAGAGCGTC	
B	MHV 1b – MHV S	1173	GACTTAGTCCTCTCCTTGATTG	2479
		1127	CCAGTAAGCAATAATGTGG	
C	IBV 1b – IBV S	IBV.F73	TCAGCATGGACGTGTGGTTA	992
		IBV.R73	CCCCATGTAAATGCCAACCA	
D	IBV S – IBV S	IBV.F14	TAAATGGTGATCTTGTTT	708
		IBV.R13	CGCTCTTAGTAACATAAAC	
E	IBV S – IBV M	IBV.F15	TGCTGCTTCCTTTAATAAG	1994
		IBV.R15	CTGCGACAAGACCTCCTG	
F	IBV 1b – MHV S	mIBV.F47	TCAGCATGGACGTGTGGTTA	1035
		mIBV.R47	CCCAGGCCTTGTGAAACTTC	
G	MHV S – MHV S	MHV.F05	ACCCTCCGCTACTACGTTTT	966
		MHV.R05	AGGCAGGTATCATGTGACCA	
H	MHV S – IBV M	MHV.F08	GGATGGGTTTGATGCAACCA	2129
		IBV.R37	GAGAAAGCACCATTGGCACA	

Primer set letters refer to Figs. 1 and 3

### Targeted RNA recombination and rescue of recombinant IBV

Recombinant IBV (rIBV) was generated by substituting the IBV spike ectodomain back into the mIBV genome by targeted RNA recombination between p-IBV-generated donor RNA and recipient virus mIBV. LR7 cells were infected with mIBV at a multiplicity of infection (MOI) of 0.4 for 4 h. Capped, run-off donor transcripts from p-IBV were transfected into the mIBV-infected LR7 cells by electroporation with two pulses at 850 V and 50 μF. Electroporated LR7 cells were resuspended in 2 ml DMEM (at 37 °C) and tenfold dilutions (up to 10^−3^) were prepared. Two hundred microliters of LR7 cell suspensions were inoculated into the allantoic cavity of 10-day-old ECEs, using 5 eggs per dilution. The eggs were candled twice daily and scored for embryonic death. Upon death, or at 7 days p.i., the eggs were transferred to 4 °C. Sixteen to twenty-four hrs later the AF was collected aseptically for RT-qPCR, and the CAMs were fixed in 10% formalin for immunohistochemistry (IHC). The AF from eggs inoculated with the highest dilution of electroporated LR7 cells, in which virus was detected by RT-qPCR and IHC, was subjected to two additional rounds of end-point dilution in 8-day-old ECE. Genetic characterization of candidate recombinants was performed by RT-PCR and subsequent Sanger sequencing of the region encoding the structural and accessory genes using the primer sets specified in Table 3.

### Immunofluorescence staining

Biological characterization of the chimeric nature of mIBV was performed by immunofluorescence (IF) double staining for IBV and MHV. BHK-21 and LR7 cells were grown on coverslips and inoculated with IBV Beaudette, and MHV A59 and mIBV #1B3-IIA, respectively, at an MOI of 1.0. Cells were fixed with PBS 4% paraformaldehyde (Aurion, Wageningen, The Netherlands) for 20 min at room temperature after 5¼, 8, and 10 hpi for MHV, IBV, and mIBV, respectively. Subsequently, cells were permeabilized with PBS containing 0.1% Triton X-100, blocked with goat serum (Gibco by Life Technologies), and incubated for 45–60 min with a combination of two primary antibodies in NGS; rabbit anti-MHV polyserum k134 diluted 1:400 and chicken anti-IBV-H120 serum diluted 1:400, or rabbit anti-MHV polyserum k134 diluted 1:400 and mouse MAb Ch/IBV 26.1 diluted 1:200. Cells were washed three times with PBS/0.05% Tween-20 and incubated in the dark for 45 min with a combination of two fluorescently labelled secondary antibodies diluted 1:200 in NGS: Alexa Fluor 488 goat anti-chicken IgY and Alexa Fluor 568 goat anti-rabbit IgG, or Alexa Fluor 488 goat anti-mouse IgG and Alexa Fluor 568 goat anti-rabbit IgG. Cells were washed three times and nuclei were stained with 300 nM DAPI in PBS for 5–10 min in the dark. Cells were washed once with milliQ and mounted with Fluorsave (Calbiochem by Merck Millipore, Billerica, MA, USA). Slides were viewed using an Olympus BX60 microscope with filters I3, A and N2.1 with a Leica DFC425C color CCD and Leica LAS-AF software (Leica Microsystems, Wetzlar, Germany).

### In ovo kinetics of IBV and rIBV-wt

Eight-day-old ECEs were inoculated with 10^2^ EID_50_ of IBV H52 BI or recombinant IBV wild-type (rIBV-wt). Eggs were candled twice daily and 6, 12, 24, 36, and 48 hpi. Five previously selected eggs per virus strain were transferred to 4 °C for 16–24 h, and AF was aseptically harvested and stored at −80 °C. For analysis, AF samples were thawed and tenfold diluted in PBS without Ca and Mg, and nucleic acids were extracted with the QIAamp DNA Blood Mini kit (Qiagen, Hilden, Germany) and the addition of carrier RNA, using the Hamilton Starlet pipet robot (Reno, Nevada, USA). Extracted nucleic acids were analyzed by RT-qPCR for the amount of IBV RNA following the protocol from Callison et al. [42] with small adaptations. Briefly, the same primers and probe were applied, while the thermoprofile was adapted for use of the ABI TaqMan Fast Virus 1-Step Master Mix (Applied Biosystems by Thermo Fisher Scientific) and the Roche 480 LightCycler. All nucleic acid samples were run and analyzed in triplicates using a tenfold dilution series of IBV H52 BI as reference for quantification. The embryonic survival at each of the time points was calculated according to the number of embryos alive at each time point compared to the total number of animals still in the experiment. A paired t-test was performed to analyse the differences in embryonic death between IBV H52 BI and rIBV-wt.

### In vivo characteristics of IBV H52 BI and rIBV-wt

SPF layer-type chickens were used in the experimental infection study at Boehringer Ingelheim Animal Health Operations (BIAHO, Weesp, The Netherlands). The chickens were hatched from SPF eggs at BIAHO from eggs obtained from Charles River (BIOVO Kft, Mohács, Magyarország, Hungary). One-day-old chickens were kept in separate isolators under controlled housing conditions, including filtered supply and exhaust air.

Animals were housed in separate groups and inoculated via eye-drop with 10^3^ EID_50_ in 0.1 ml of IBV H52 BI (*n* = 5), rIBV-wt (*n* = 5), or not inoculated (*n* = 5, negative control). Clinical symptoms monitored included ruffled feathers, decreased consciousness, depression, gasping, coughing, tracheal rales, and nasal discharge. Seven days p.i. animals were euthanized, and evaluated for their tracheal ciliary activity. To this end, the trachea was sliced into 10 transversal sections: 3 from the upper part, 4 from the middle part, and 3 from the lower part. Ciliary activity was examined by low-magnification microscopy within 2 h after sampling. Ciliostasis of each tracheal section was scored on a scale from 0 (100% ciliary activity) to 4 (no ciliary activity, i.e. complete ciliostasis), with the maximum score for each trachea being 40. Finally, the mean ciliostasis score for each group of animals was calculated. A unpaired t-test was performed to analyse whether there were differences in ciliostasis scores between IBV H52 BI and rIBV-wt.

## Results

### Generation and antigenic characterization of mIBV and rIBV-wt

Viral RNA of IBV H52 BI and RNAs transcribed from plasmids p-mIBV and p-IBV-N were co-transfected into BHK-21 cells and seeded onto monolayers of LR7 cells. At 2 days post transfection, syncytia were observed in the LR7 monolayers, suggesting the successful generation of recombinant mIBV. After two rounds of plaque purification on LR7 cells, candidate recombinants were characterized antigenically and genetically.

**Fig. 2:**
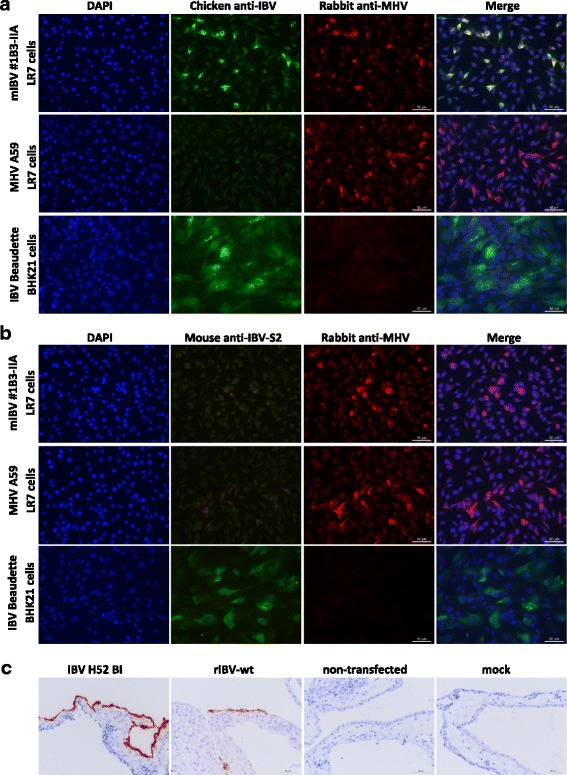
Antigenic characterization of mIBV and rIBV-wt. **a** Immunofluorescence analyses of IBV Beaudette, MHV A59 and mIBV #1B3-IIA infected cells. LR7 cells infected with mIBV were fixed and double-immunolabeled with a polyclonal against IBV (*green*) and a polyclonal antibody against MHV (red). IBV Beaudette-infected BHK-21 cells and MHV-infected LR7 cells were taken along for comparison. Nuclei are visualized with DAPI (*blue*). Overlay pictures (Merge) are shown on the right. **b** Similar to (**a**), except that a monoclonal antibody against IBV S2 was used instead of a polyclonal against IBV, indicating the absence of IBV S2 protein in mIBV infected cells. **c** Immunohistochemistry of IBV H52 BI and rIBV-wt infected CAM tissues. Ten-day-old embryonated chicken eggs were inoculated with IBV H52 BI (positive control), mIBV-infected and p-IBV transcript electroporated LR7 cells (resulting in generation of rIBV-wt), mIBV infected and p-IBV transcript, but not electroporated, LR7 cells (mIBV + p-IBV mock) or PBS (mock). Formalin-fixed and paraffin-embedded CAM tissues were immunohistochemically stained using a monoclonal antibody against IBV S2. Replication of (r)IBV in the epithelial cells of the CAM is indicated by red cytoplasmic staining, which is absent in eggs inoculated with mIBV-infected non-transfected LR7 cells

IF staining of LR7 cells infected with mIBV showed positive staining with both anti-IBV and anti-MHV sera, indicating the chimeric nature of mIBV (Fig. 2a). IF staining with an anti-IBV-S2 MAb was positive for IBV Beaudette-infected BHK-21 cells (taken along as positive control for IF), but not for LR7 cells infected with mIBV, indicating the absence of IBV S protein in mIBV (Fig. 2b).

LR7 cells infected with mIBV and subsequently transfected with RNA transcribed from plasmid p-IBV were inoculated in tenfold dilution series into the allantoic cavity of 10-day-old ECEs. No embryonic death was observed up to 7 days p.i., but embryos in the lowest dilutions showed signs of stunting and curling typical for embryos infected with IBV. The presence of replicating recombinant IBV was demonstrated by RT-qPCR on viral RNA extracted from the AF (data not shown), and by IHC on CAM tissue (Fig. 2c). In contrast, CAMs of eggs inoculated with mIBV-infected LR7 cells (not transfected) did not show any positive signal for IBV in IHC. During the first and second passage of rIBV-wt in eight-day-old ECEs for end-point dilution purposes, infected embryos died between 2 and 3 days p.i.

### Genetic characterization of mIBV and rIBV-wt

**Fig. 3:**
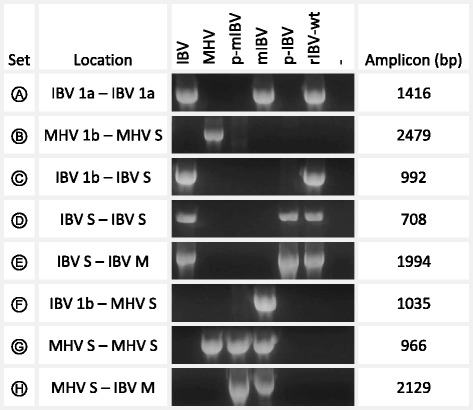
Genetic characterization of wild type viruses, recombinant viruses and donor plasmids. PCR was performed on cDNA templates of viral RNA extracted from infected LR7 cell culture supernatants (MHV and mIBV) and allantoic fluid of inoculated embryonated eggs (IBV and rIBV-wt), plasmid DNA (p-mIBV and p-IBV), or no template control (−).Primer set letters A to H correspond with letters depicted in Fig. 1; detailed information on the primers is given in Table 3

Using specifically located primers (Fig. 1 and Table 3), the intended genome structure and sequence of mIBV and recombinant IBV wild-type (rIBV-wt) were verified by RT-PCR (Fig. 3). The most important findings were that (1) murinized IBV contained the correct 5′ S gene sequence (Primer set [F] - with a forward primer located in the IBV 1b gene at a position upstream of the 1b sequence present in p-mIBV and a reverse primer located in the MHV spike gene - resulted in a detectable PCR product for mIBV, but not for IBV or p-mIBV); (2) murizined IBV contained the MHV spike gene at the location of the IBV spike gene (as both primer set [F] and [H] - each with a primer in the MHV spike gene and one in IBV gene 1b [F] or in the M gene [H] - resulted in a detectable PCR product for mIBV, but not for IBV or MHV); (3) the IBV spike gene was absent from the mIBV genome (as primer set [D] targeting the IBV spike gene resulted in a detectable PCR product for IBV, but not for mIBV or MHV(4); recombinant IBV was the result of recombination between genomic RNA from mIBV and RNA transcribed from p-IBV (as primer set [C] - with a forward primer in IBV gene 1b located upstream of the 1b sequence present in p-mIBV and a reverse primer located in the IBV spike gene - resulted in a detectable PCR product for rIBV-wt, but not for mIBV or p-IBV); (5) recombinant IBV contained the IBV spike gene at the location of the MHV-derived spike in mIBV (as both primer set [C] and primer set [E] - each with one primer in the IBV spike gene and the other in IBV ORF 1b [C] or the M gene [E] - resulted in a detectable PCR product for rIBV-wt, but not for mIBV or MHV); (6) the MHV spike gene was absent from the rIBV-wt genome (as primer set [G] targeting the MHV spike gene resulted in a detectable PCR product for MHV and mIBV, but not for rIBV-wt).

Sequence analysis of the 3′ 9 kb of the mIBV genome (starting 1 kb upstream of the *Bst*BI RES in gene 1b, which marks the start of p-mIBV) confirmed the expected genetic identity of mIBV as observed after RT-PCR analysis (Additional file 2: Figure S1). The 3′ 9 kb of mIBV and rIBV-wt were exactly as designed, including the deliberate synonymous mutations listed in Additional file 1: Table S1. A single spontaneous silent mutation (T to C) was observed in the spike of rIBV-wt at position 22,644.

### Recombinant IBV growth kinetics in embryonated eggs

**Fig. 4:**
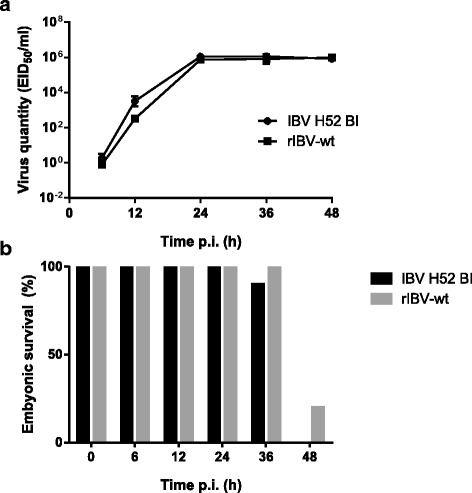
*In ovo* characteristics of IBV and rIBV-wt. **a** Growth kinetics of IBV and rIBV-wt were assessed by quantitative RT-qPCR analysis of RNA extracted from allantoic fluid of inoculated embryonated eggs collected at 6, 12, 24, 36 and 48 hpi. Data points represent means and standard deviations of 5 eggs per condition, with all samples run and analyzed in triplicates, using a tenfold dilution series of IBV H52 BI as reference for quantification. **b** Embryonic death is indicated as a percentage of all remaining animals at each time point

*In ovo* growth kinetics of IBV and rIBV-wt were assessed after inoculating ECEs with 10^2^ EID_50_ per egg by determination of the relative viral load in the AF of five eggs per virus at 6, 12, 24, 36 and 48 hpi by RT-qPCR. At 12 hpi, the parental wild-type IBV showed somewhat higher viral loads as compared to rIBV-wt, while from 24 hpi onwards, viral loads were comparable for both viruses (Fig. 4a). The virus titers remained at the same level until embryos started to die between 36 and 48 hpi. No differences in embryonic death between rIBV-wt and IBV H52 BI groups was observed (Fig. 4b; *p* > 0.05).

### In vivo characteristics of rIBV-wt

**Fig. 5:**
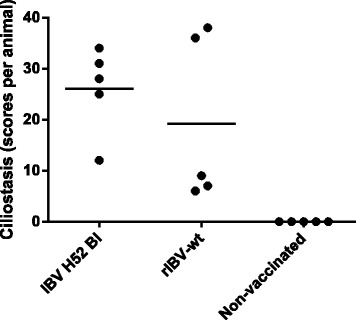
In vivo pathogenicity of IBV and rIBV-wt. Tracheal ciliostasis scores per individual animal and means per experimental group are plotted. Maximal ciliostasis score per animal is 40, which indicates complete ciliostasis in all 10 transversal tracheal sections examined. Ciliostasis as determined at 7 days p.i. with vaccine strain IBV H52 BI, rIBV-wt or non-vaccinated (5 animals per group)

The pathogenicity of rIBV-wt was compared to that of the parental IBV H52 BI strain by inoculating one-day-old SPF chickens. During the course of the infection, no clinical symptoms were observed in any of the groups (data not shown). At 7 days p.i. the animals were euthanized and the mean ciliostasis scores were determined as a readout for the ability of the respective viruses to infect and cause lesions in the primary target organ, the trachea. As expected, the negative control animals scored very low (Fig. 5), while IBV H52 BI infection resulted in a score of 26. rIBV-wt had a mean score of 19 (*P* > 0.05), indicating that rIBV maintained the ability to infect one-day-old chickens and induced lesions to a similar extent as the parental virus strain.

## Discussion

Here we developed a novel RGS based on targeted RNA recombination, which allows manipulation of the genome of virulent IBV. The resulting recombinant virus has the same characteristics as the wild type IBV H52 both in embryonated eggs and in one-day-old chickens.

By adapting the classical targeted RNA recombination approach [11, 19, 33] to IBV, the inability to culture IBV strains like H52 on continuous cell lines has been overcome. For this, a cell-line known to support the replication, but not the entry of, IBV was used. This observation was used to create, by co-transfection of IBV viral RNA and a transcript of chimeric IBV carrying the MHV spike gene, the recombinant mIBV virus. Upon transfection of synthetic rIBV donor RNA into mIBV-infected murine LR7 cells, subsequent infectious IBV virus particles could be rescued in ECE. The feasibility of the approach was demonstrated by the generation of recombinant rIBV virus carrying silent marker mutations.

The inability to select for individual IBV recombinants by plaque purification was circumvented by a combination of three approaches. First, the mIBV-infected and rIBV donor RNA-transfected LR7 cells were inoculated into ECEs by end-point dilution. Second, early RT-PCR and sequencing based screening of the genetic make-up of recombinants helped to identify and discard erroneous recombinants. Third, two subsequent end-point dilution series were executed in ECEs, each leading to the selection of rIBV-wt. Finally, the genetic identity of the 5′ 9 kb of rIBV-wt, i.e. the part of the IBV genome had been subject to manipulation, was confirmed by sequence analysis.

The replication and pathogenicity of rIBV-wt in ECE was comparable to that of the parental IBV H52 BI. Viral loads in the AF were similar with respect to maximum virus titers (Fig. 4) and embryonic death induced by both viruses did not differ (not shown). The pathogenicity of rIBV-wt in one-day-old chickens was also comparable to that of parental IBV H52 BI, as demonstrated by comparable mean ciliostasis scores at 7 days p.i. (Fig. 5). Taken together, rIBV-wt has the same properties as IBV H52 both *in ovo* and in vivo and can thus be used to provide insights in the infection process in chickens. Previously described RGS based on non-pathogenic IBV [21–25] can be used for in vivo studies only upon introduction of virulence factors including spikes from other IBV serotypes [26–28]. Our newly developed RGS for IBV-H52 directly allows the elucidation of factors that determine the pathogenicity of IBV, as well as studying its protective immunity in vivo.

Here, for the first time host species switching of an avian gammacoronavirus to mammalian cells was demonstrated, by exchanging the spike gene with that of the betacoronavirus MHV. Manipulation of coronavirus genomes by targeted RNA recombination using an interspecies chimeric coronavirus has been demonstrated for alpha- and betacoronaviruses, thereby switching species tropism between mammalian hosts. Our observation confirms the spike gene as the principal determinant of host species tropism of both avian and mammalian coronaviruses.

## Conclusion

In summary, a novel reverse genetics system (RGS) based on targeted RNA recombination that allowed manipulation of the genome of virulent IBV was developed. This system makes use of an interspecies chimeric coronavirus, which is created by replacing the ectodomain of the IBV spike protein by that of MHV. The spike ectodomain exchange results in a host species tropism switch, which enables replication of mIBV in cell culture. Upon recombination of mIBV with synthetic donor RNA carrying the IBV spike gene, rIBV could be rescued in ECEs. *In ovo* growth kinetics and in vivo characteristics were comparable for rIBV-wt and parental IBV H52 BI, suggesting no attenuating effect of the recombination process or of the introduced synonymous marker mutations. This system will allow the introduction of mutations in the 3′ one-third of the IBV genome, allowing the manipulation of the structural and accessory genes. The use of this system for both fundamental and applied research is promising, and potentially enables the development of a new generation of rationally designed live-attenuated IBV vaccines.

## Additional files

Additional file 1: Table S1. Silent mutations introduced in rIBV. Nucleotide positions and sequences refer to the IBV H52 BI genome (see Additional file 2: Figure S1). Modified nucleotides in recombinant IBV wt are depicted in lower case; n.a. = not applicable. Purpose of introduction of restriction enzyme site are indicated for each site; in case of enzyme site removal the purpose was to create unique restriction enzyme sites for cloning; n.a. = not applicable in this study. (DOCX 19 kb)

Additional file 2: Figure S1. Alignment of 3′ 9 kb of mIBV and rIBV-wt with IBV H52 BI. Alignment of the 3′ 9 kb of mIBV 1B3IIA P6 (excluding the MHV derived spike ectodomain sequence) and recombinant (r)IBV wild-type (wt) P4 with IBV H52 BI. Numbers refer to nucleotide positions in the IBV H52 BI genome. Restriction enzyme sites are highlighted in yellow, with the corresponding enzyme indicated above the sequences. An additional thymidine residue to keep the MHV spike gene ectodomain sequence in frame with the IBV spike gene signal sequence at position 20,385 is highlighted in green and marked with a # above the sequence. A spontaneous T to C silent substitution in the spike of rIBV-wt at position 22,644 is highlighted in red. (DOCX 41 kb)

## Abbreviations

AF: Allantoic fluid
CAM: Chorio-allantoic membrane
ECE: Embryonated chicken eggs
EID: Embryonic infectious dose
hpi: Hours post infection
IBV: Infectious bronchitis virus
MHV: Mouse hepatitis virus
mIBV: Murinized infectious bronchitis virus
RES: Restriction enzyme sites
RGS: Reverse genetics system
rIBV: Recombinant infectious bronchitis virus
SPF: Specific pathogen free
Wt: Wild type
