# Supplementary material for: A reverse genetics system for avian coronavirus infectious bronchitis virus based on targeted RNA recombination
Source: Virol J. 2017 Jun 12;14:109. doi: 10.1186/s12985-017-0775-8 (PMC5468965; doi:10.1186/s12985-017-0775-8)
Supplement: Supplementary file 1 — Silent mutations introduced in rIBV. Nucleotide positions and sequences refer to the IBV H52 BI genome (see Additional file 2: Figure S1). Modified nucleotides in recombinant IBV wt are depicted in lower case; n.a. = not applicable. Purpose of introduction of restriction enzyme site are indicated for each site; in case of enzyme site removal the purpose was to create unique restriction enzyme sites for cloning; n.a. = not applicable in this study. (DOCX 19 kb) [file 12985_2017_775_MOESM1_ESM.docx]

**Additional file 1: Table S1.** Silent mutations introduced in rIBV

| **Gene** | **Nucleotide positions** | **IBV H52 BI sequence** | **rIBV-wt sequence** | **Restriction enzyme** | **Function** |
| --- | --- | --- | --- | --- | --- |
| 1b | 20247..20252 | TTGAAA | cTtAAg | AflII | n.a. |
| 1b | 20283..20290 | TTAATTAA | TTgATaAA | PacI | Removed |
| S | 20379..20384 | CAGTAG | ctcgAG | XhoI | Exchange S |
| S | 21356..21361 | TCAATTG | agtATTG | MfeI | Removed |
| S | 21636..21641 | CCAAGG | CCAgGG | StyI | Removed |
| S | 21995..22000 | TCAATTG | agtATTG | MfeI | Removed |
| S | 22018..22023 | CAATTG | CAgTTa | MfeI | Removed |
| S | 22114..22119 | ACGCGT | ACtCGg | MluI | Removed |
| S | 22480..22485 | CTTAAG | CTgAAa | AflII | Removed |
| S | 22564..22569 | ACTAGT | ACaAGc | SpeI | Removed |
| S | 23604..23609 | GTTAGC | GcTAGC | NheI | n.a. |
| 3a | 23817..23822 | ACGTCC | ACtagt | SpeI | n.a. |
| 3a | 23888..23893 | AGCGCT | tGCcCT | AfeI | Removed |
| 3b | 24002..24007 | ACTGGT | ACcGGT | AgeI | n.a. |
| E | 24200..24205 | AGCGCT | gGCaCT | AfeI | Removed |
| E | 24299..24304 | TACATG | cACgTG | PmlI | n.a. |
| M | 24574..24579 | ACAAGA | ACgcGt | MluI | n.a. |
| IR | 25467..25472 | AGCGCT | n.a. | AfeI | n.a. |
| 5a | 25500..25505 | ACTAGT | n.a. | SpeI | n.a. |
| 5a | 25595..25600 | ACGCGT | gCGaGT | MluI | Removed |
| 5b | 25825..25830 | CCAAGG | tCAgGG | StyI | Removed |
| N | 25969..25974 | GCATCT | GCtagc | NheI | n.a. |
| N | 26792..26797 | CACGTG | CtaGgG | PmlI | Removed |
| N | 26857..26862 | ACGCGT | ACtCGg | MluI | Removed |
| N | 27053..27058 | CCAAGG | CgAAaG | StyI | Removed |
