# Supplementary material for: A reverse genetics system for avian coronavirus infectious bronchitis virus based on targeted RNA recombination
Source: Virol J. 2017 Jun 12;14:109. doi: 10.1186/s12985-017-0775-8 (PMC5468965; doi:10.1186/s12985-017-0775-8)
Supplement: Supplementary file 2 — Alignment of 3′ 9 kb of mIBV and rIBV-wt with IBV H52 BI. Alignment of the 3′ 9 kb of mIBV 1B3IIA P6 (excluding the MHV derived spike ectodomain sequence) and recombinant (r)IBV wild-type (wt) P4 with IBV H52 BI. Numbers refer to nucleotide positions in the IBV H52 BI genome. Restriction enzyme sites are highlighted in yellow, with the corresponding enzyme indicated above the sequences. An additional thymidine residue to keep the MHV spike gene ectodomain sequence in frame with the IBV spike gene signal sequence at position 20,385 is highlighted in green and marked with a # above the sequence. A spontaneous T to C silent substitution in the spike of rIBV-wt at position 22,644 is highlighted in red. (DOCX 41 kb) [file 12985_2017_775_MOESM2_ESM.docx]

**Additional file 2: Figure S1.** Alignment of last 9 kb of mIBV and rIBV-wt with IBV H52 BI

18612

IBV_H52_BI TATGCGAAGAGAAATATTCGCACACTGCCAAATAATCGTATTCTTAAGGG

rIBV-wt_P4_1764-1773 TATGCGAAGAGAAATATTCGCACACTGCCAAATAATCGTATTCTTAAGGG

mIBV_1B3IIA_P6_excl_S_ed TATGCGAAGAGAAATATTCGCACACTGCCAAATAATCGTATTCTTAAGGG

**************************************************

18662

IBV_H52_BI TCTTGGTGTAGACGTAACTAATGGATTTGTAATTTGGGACTACACGAACC

rIBV-wt_P4_1764-1773 TCTTGGTGTAGACGTAACTAATGGATTTGTAATTTGGGACTACACGAACC

mIBV_1B3IIA_P6_excl_S_ed TCTTGGTGTAGACGTAACTAATGGATTTGTAATTTGGGACTACACGAACC

**************************************************

18712

IBV_H52_BI AGACACCATTATATCGTAATACTGTTAAGGTATGTGCATACACAGACATT

rIBV-wt_P4_1764-1773 AGACACCATTATATCGTAATACTGTTAAGGTATGTGCATACACAGACATT

mIBV_1B3IIA_P6_excl_S_ed AGACACCATTATATCGTAATACTGTTAAGGTATGTGCATACACAGACATT

**************************************************

18762

IBV_H52_BI GAGCCAAATGGCCTAATAGTTCTGTATGATGATAGATATGGTGATTACCA

rIBV-wt_P4_1764-1773 GAGCCAAATGGCCTAATAGTTCTGTATGATGATAGATATGGTGATTACCA

mIBV_1B3IIA_P6_excl_S_ed GAGCCAAATGGCCTAATAGTTCTGTATGATGATAGATATGGTGATTACCA

**************************************************

18812

IBV_H52_BI ATCTTTTCTTGCCGCTGATAATGCTGTTCTAGTTTCTACACAGTGTTATA

rIBV-wt_P4_1764-1773 ATCTTTTCTTGCCGCTGATAATGCTGTTCTAGTTTCTACACAGTGTTATA

mIBV_1B3IIA_P6_excl_S_ed ATCTTTTCTTGCCGCTGATAATGCTGTTCTAGTTTCTACACAGTGTTATA

**************************************************

18862

IBV_H52_BI AGCGATATTCATATGTAGAAATACCGTCAAATATGCTTGTTCAGAATGGT

rIBV-wt_P4_1764-1773 AGCGATATTCATATGTAGAAATACCGTCAAATATGCTTGTTCAGAATGGT

mIBV_1B3IIA_P6_excl_S_ed AGCGATATTCATATGTAGAAATACCGTCAAATATGCTTGTTCAGAATGGT

**************************************************

18912

IBV_H52_BI ATGCCATTAAAAGATGGAGCGAATCTGTATGTTTATAAGCGCGTTAATGG

rIBV-wt_P4_1764-1773 ATGCCATTAAAAGATGGAGCGAATCTGTATGTTTATAAGCGCGTTAATGG

mIBV_1B3IIA_P6_excl_S_ed ATGCCATTAAAAGATGGAGCGAATCTGTATGTTTATAAGCGCGTTAATGG

**************************************************

18962

IBV_H52_BI TGCGTTTGTTACGCTACCCAATACATTAAACACACAGGGCCGCAGTTATG

rIBV-wt_P4_1764-1773 TGCGTTTGTTACGCTACCCAATACATTAAACACACAGGGCCGCAGTTATG

mIBV_1B3IIA_P6_excl_S_ed TGCGTTTGTTACGCTACCCAATACATTAAACACACAGGGCCGCAGTTATG

**************************************************

19012

IBV_H52_BI AAACTTTTGAACCTCGTAGTGACGTTGAGCGTGATTTTCTCGACATGTCA

rIBV-wt_P4_1764-1773 AAACTTTTGAACCTCGTAGTGACGTTGAGCGTGATTTTCTCGACATGTCA

mIBV_1B3IIA_P6_excl_S_ed AAACTTTTGAACCTCGTAGTGACGTTGAGCGTGATTTTCTCGACATGTCA

**************************************************

19062

IBV_H52_BI GAGGAGGATTTTGTAGAAAAGTATGGTAAAGACTTAGGTCTACAACACAT

rIBV-wt_P4_1764-1773 GAGGAGGATTTTGTAGAAAAGTATGGTAAAGACTTAGGTCTACAACACAT

mIBV_1B3IIA_P6_excl_S_ed GAGGAGGATTTTGTAGAAAAGTATGGTAAAGACTTAGGTCTACAACACAT

**************************************************

19112

IBV_H52_BI ACTGTATGGTGAAGTTGATAAACCACAATTGGGCGGTTTACACACTGTTA

rIBV-wt_P4_1764-1773 ACTGTATGGTGAAGTTGATAAACCACAATTGGGCGGTTTACACACTGTTA

mIBV_1B3IIA_P6_excl_S_ed ACTGTATGGTGAAGTTGATAAACCACAATTGGGCGGTTTACACACTGTTA

**************************************************

19162

IBV_H52_BI TAGGTATGTACAGACTTTTACGTGCGAATAAGTTGAATGCAAAGTCTGTT

rIBV-wt_P4_1764-1773 TAGGTATGTACAGACTTTTACGTGCGAATAAGTTGAATGCAAAGTCTGTT

mIBV_1B3IIA_P6_excl_S_ed TAGGTATGTACAGACTTTTACGTGCGAATAAGTTGAATGCAAAGTCTGTT

**************************************************

19212

IBV_H52_BI ACTAATTCAGATTCTGATGTCATGCAAAATTATTTTGTGTTGGCAGACAA

rIBV-wt_P4_1764-1773 ACTAATTCAGATTCTGATGTCATGCAAAATTATTTTGTGTTGGCAGACAA

mIBV_1B3IIA_P6_excl_S_ed ACTAATTCAGATTCTGATGTCATGCAAAATTATTTTGTGTTGGCAGACAA

**************************************************

19262

IBV_H52_BI TGGTTCTTACAAGCAAGTGTGCACTGTTGTGGATTTACTGCTTGATGATT

rIBV-wt_P4_1764-1773 TGGTTCTTACAAGCAAGTGTGCACTGTTGTGGATTTACTGCTTGATGATT

mIBV_1B3IIA_P6_excl_S_ed TGGTTCTTACAAGCAAGTGTGCACTGTTGTGGATTTACTGCTTGATGATT

**************************************************

19312

IBV_H52_BI TCTTAGAACTTCTTAGGAACATACTGAATGAGTATGGTACTAATAAGTCA

rIBV-wt_P4_1764-1773 TCTTAGAACTTCTTAGGAACATACTGAATGAGTATGGTACTAATAAGTCA

mIBV_1B3IIA_P6_excl_S_ed TCTTAGAACTTCTTAGGAACATACTGAATGAGTATGGTACTAATAAGTCA

**************************************************

19362

IBV_H52_BI AAAGTTGTAACAGTGTCAATTGATTACCATAGCATAAATTTTATGACTTG

rIBV-wt_P4_1764-1773 AAAGTTGTAACAGTGTCAATTGATTACCATAGCATAAATTTTATGACTTG

mIBV_1B3IIA_P6_excl_S_ed AAAGTTGTAACAGTGTCAATTGATTACCATAGCATAAATTTTATGACTTG

**************************************************

19412

IBV_H52_BI GTTTGAAGATGGCAGTATTAAAACATGTTACCCACAGCTTCAATCAGCAT

rIBV-wt_P4_1764-1773 GTTTGAAGATGGCAGTATTAAAACATGTTACCCACAGCTTCAATCAGCAT

mIBV_1B3IIA_P6_excl_S_ed GTTTGAAGATGGCAGTATTAAAACATGTTACCCACAGCTTCAATCAGCAT

**************************************************

19462

IBV_H52_BI GGACGTGTGGTTATAATATGCCTGAACTCTATAAAGTCCAGAATTGTGTT

rIBV-wt_P4_1764-1773 GGACGTGTGGTTATAATATGCCTGAACTCTATAAAGTCCAGAATTGTGTT

mIBV_1B3IIA_P6_excl_S_ed GGACGTGTGGTTATAATATGCCTGAACTCTATAAAGTCCAGAATTGTGTT

**************************************************

19512

IBV_H52_BI ATGGAACCTTGCAACATTCCTAATTATGGTATTGGAATAACGTTGCCAAG

rIBV-wt_P4_1764-1773 ATGGAACCTTGCAACATTCCTAATTATGGTATTGGAATAACGTTGCCAAG

mIBV_1B3IIA_P6_excl_S_ed ATGGAACCTTGCAACATTCCTAATTATGGTATTGGAATAACGTTGCCAAG

**************************************************

19562

IBV_H52_BI TGGTATTATGATGAATGTGGCAAAGTACACACAACTTTGTCAGTACCTTT

rIBV-wt_P4_1764-1773 TGGTATTATGATGAATGTGGCAAAGTACACACAACTTTGTCAGTACCTTT

mIBV_1B3IIA_P6_excl_S_ed TGGTATTATGATGAATGTGGCAAAGTACACACAACTTTGTCAGTACCTTT

**************************************************

19612

IBV_H52_BI CGAAAACAACAATGTGTGTGCCGCATAATATGCGCGTTATGCATTTTGGA

rIBV-wt_P4_1764-1773 CGAAAACAACAATGTGTGTGCCGCATAATATGCGCGTTATGCATTTTGGA

mIBV_1B3IIA_P6_excl_S_ed CGAAAACAACAATGTGTGTGCCGCATAATATGCGCGTTATGCATTTTGGA

**************************************************

19662

IBV_H52_BI GCAGGTAGTGATAAAGGAGTGGCGCCTGGTAGTACTGTTCTTAAACAGTG

rIBV-wt_P4_1764-1773 GCAGGTAGTGATAAAGGAGTGGCGCCTGGTAGTACTGTTCTTAAACAGTG

mIBV_1B3IIA_P6_excl_S_ed GCAGGTAGTGATAAAGGAGTGGCGCCTGGTAGTACTGTTCTTAAACAGTG

**************************************************

19712

IBV_H52_BI GCTCCCCGAAGGAACACTCCTTGTCGATAATGATATTGTAGATTATGTTT

rIBV-wt_P4_1764-1773 GCTCCCCGAAGGAACACTCCTTGTCGATAATGATATTGTAGATTATGTTT

mIBV_1B3IIA_P6_excl_S_ed GCTCCCCGAAGGAACACTCCTTGTCGATAATGATATTGTAGATTATGTTT

**************************************************

19762

IBV_H52_BI CTGACGCACACGTTTCTGTGCTTTCAGATTGCAATAAATATAAGACAGAG

rIBV-wt_P4_1764-1773 CTGACGCACACGTTTCTGTGCTTTCAGATTGCAATAAATATAAGACAGAG

mIBV_1B3IIA_P6_excl_S_ed CTGACGCACACGTTTCTGTGCTTTCAGATTGCAATAAATATAAGACAGAG

**************************************************

19812

IBV_H52_BI CACAAGTTTGATCTTGTGATATCTGATATGTATACAGACAATGATTCAAA

rIBV-wt_P4_1764-1773 CACAAGTTTGATCTTGTGATATCTGATATGTATACAGACAATGATTCAAA

mIBV_1B3IIA_P6_excl_S_ed CACAAGTTTGATCTTGTGATATCTGATATGTATACAGACAATGATTCAAA

**************************************************

19862

IBV_H52_BI AAGAAAGCATGAAGGCGTGATAGCCAATAATGGCAATGATGACGTCTTCA

rIBV-wt_P4_1764-1773 AAGAAAGCATGAAGGCGTGATAGCCAATAATGGCAATGATGACGTCTTCA

mIBV_1B3IIA_P6_excl_S_ed AAGAAAGCATGAAGGCGTGATAGCCAATAATGGCAATGATGACGTCTTCA

**************************************************

19912

IBV_H52_BI TATACCTTTCAAGTTTTCTACGCAATAATTTGGCTCTGGGAGGCAGTTTT

rIBV-wt_P4_1764-1773 TATACCTTTCAAGTTTTCTACGCAATAATTTGGCTCTGGGAGGCAGTTTT

mIBV_1B3IIA_P6_excl_S_ed TATACCTTTCAAGTTTTCTACGCAATAATTTGGCTCTGGGAGGCAGTTTT

**************************************************

19962

IBV_H52_BI GCTGTAAAATTAACAGAGACAAGTTGGCATGAGAGTTTATATGACATTGC

rIBV-wt_P4_1764-1773 GCTGTAAAATTAACAGAGACAAGTTGGCATGAGAGTTTATATGACATTGC

mIBV_1B3IIA_P6_excl_S_ed GCTGTAAAATTAACAGAGACAAGTTGGCATGAGAGTTTATATGACATTGC

**************************************************

20012

IBV_H52_BI ACAGGATTGTGCATGGTGGACAATGTTTTGTACAGCAGTGAATGCATCTT

rIBV-wt_P4_1764-1773 ACAGGATTGTGCATGGTGGACAATGTTTTGTACAGCAGTGAATGCATCTT

mIBV_1B3IIA_P6_excl_S_ed ACAGGATTGTGCATGGTGGACAATGTTTTGTACAGCAGTGAATGCATCTT

**************************************************

20062

IBV_H52_BI CTTCAGAAGCATTCCTGATTGGTGTTAATTACTTGGGTGCAAGTGCAAAG

rIBV-wt_P4_1764-1773 CTTCAGAAGCATTCCTGATTGGTGTTAATTACTTGGGTGCAAGTGCAAAG

mIBV_1B3IIA_P6_excl_S_ed CTTCAGAAGCATTCCTGATTGGTGTTAATTACTTGGGTGCAAGTGCAAAG

**************************************************

20112

IBV_H52_BI GTTAAAGTTAGTGGAAAAACACTGCACGCAAATTATATATTTTGGAGGAA

rIBV-wt_P4_1764-1773 GTTAAAGTTAGTGGAAAAACACTGCACGCAAATTATATATTTTGGAGGAA

mIBV_1B3IIA_P6_excl_S_ed GTTAAAGTTAGTGGAAAAACACTGCACGCAAATTATATATTTTGGAGGAA

**************************************************

20162

IBV_H52_BI TTGTAATTATTTACAAACCTCTGCTTATAGTATATTTGATGTTGCTAAGT

rIBV-wt_P4_1764-1773 TTGTAATTATTTACAAACCTCTGCTTATAGTATATTTGATGTTGCTAAGT

mIBV_1B3IIA_P6_excl_S_ed TTGTAATTATTTACAAACCTCTGCTTATAGTATATTTGATGTTGCTAAGT

**************************************************

20212 AflII-

IBV_H52_BI TTGATTTGAGATTGAAAGCAACGCCAGTTGTTAATTTGAAAACTGAACAA

rIBV-wt_P4_1764-1773 TTGATTTGAGATTGAAAGCAACGCCAGTTGTTAATCTTAAGACTGAACAA

mIBV_1B3IIA_P6_excl_S_ed TTGATTTGAGATTGAAAGCAACGCCAGTTGTTAATCTTAAGACTGAACAA

*********************************** * ** *********

20262 --PacI--

IBV_H52_BI AAGACAGACTTAGTCTTTAATTTAATTAAGTGTGGTAAGTTACTGGTAAG

rIBV-wt_P4_1764-1773 AAGACAGACTTAGTCTTTAATTTGATAAAGTGTGGTAAGTTACTGGTAAG

mIBV_1B3IIA_P6_excl_S_ed AAGACAGACTTAGTCTTTAATTTGATAAAGTGTGGTAAGTTACTGGTAAG

*********************** ** ***********************

20312

IBV_H52_BI AGATGTTGGTAACACCTCTTTTACTAGTGACTCTTTTGTGTGCACTATGT

rIBV-wt_P4_1764-1773 AGATGTTGGTAACACCTCTTTTACTAGTGACTCTTTTGTGTGCACTATGT

mIBV_1B3IIA_P6_excl_S_ed AGATGTTGGTAACACCTCTTTTACTAGTGACTCTTTTGTGTGCACTATGT

**************************************************

20362 -XhoI-#

IBV_H52_BI AGTGCTGCTTTGTATGACAGTAGTTCTTACGTGTACTACTACCAAAGTGC

rIBV-wt_P4_1764-1773 AGTGCTGCTTTGTATGACTCGAGTTCTTACGTGTACTACTACCAAAGTGC

mIBV_1B3IIA_P6_excl_S_ed AGTGCTGCTTTGTATGACTCGAGT--------------------------

****************** ***

20412

IBV_H52_BI CTTCAGACCACCTGATGGTTGGCATTTACATGGGGGTGCGTATGCGGTTG

rIBV-wt_P4_1764-1773 CTTCAGACCACCTGATGGTTGGCATTTACATGGGGGTGCGTATGCGGTTG

mIBV_1B3IIA_P6_excl_S_ed --------------------------------------------------

20462

IBV_H52_BI TTAATATTTCTAGTGAATCTAATAATGCAGGCTCTTCATCTGGGTGTACT

rIBV-wt_P4_1764-1773 TTAATATTTCTAGTGAATCTAATAATGCAGGCTCTTCATCTGGGTGTACT

mIBV_1B3IIA_P6_excl_S_ed --------------------------------------------------

20512

IBV_H52_BI GTTGGTATTATTCATGGTGGTCGTGTTGTTAATGCTTCTTCTATAGCTAT

rIBV-wt_P4_1764-1773 GTTGGTATTATTCATGGTGGTCGTGTTGTTAATGCTTCTTCTATAGCTAT

mIBV_1B3IIA_P6_excl_S_ed --------------------------------------------------

20562

IBV_H52_BI GACGGCACCGTCATCAGGTATGGCTTGGTCTAGCAGTCAGTTTTGTACTG

rIBV-wt_P4_1764-1773 GACGGCACCGTCATCAGGTATGGCTTGGTCTAGCAGTCAGTTTTGTACTG

mIBV_1B3IIA_P6_excl_S_ed --------------------------------------------------

20612

IBV_H52_BI CATACTGTAACTTTTCAGATACTACAGTGTTTGTTACACATTGTTATAAA

rIBV-wt_P4_1764-1773 CATACTGTAACTTTTCAGATACTACAGTGTTTGTTACACATTGTTATAAA

mIBV_1B3IIA_P6_excl_S_ed --------------------------------------------------

20662

IBV_H52_BI CATGGTGGGTGTCCTATAACTGGCATGCTTCAACAGCATTCTATACGTGT

rIBV-wt_P4_1764-1773 CATGGTGGGTGTCCTATAACTGGCATGCTTCAACAGCATTCTATACGTGT

mIBV_1B3IIA_P6_excl_S_ed --------------------------------------------------

20712

IBV_H52_BI TTCTGCTATGAAAAATGGCCAGCTTTTCTATAATTTAACAGTTAGTGTAG

rIBV-wt_P4_1764-1773 TTCTGCTATGAAAAATGGCCAGCTTTTCTATAATTTAACAGTTAGTGTAG

mIBV_1B3IIA_P6_excl_S_ed --------------------------------------------------

20762

IBV_H52_BI CTAAGTACCCTACTTTTAAATCATTTCAGTGTGTTAATAATTTAACATCC

rIBV-wt_P4_1764-1773 CTAAGTACCCTACTTTTAAATCATTTCAGTGTGTTAATAATTTAACATCC

mIBV_1B3IIA_P6_excl_S_ed --------------------------------------------------

20812

IBV_H52_BI GTATATTTAAATGGTGATCTTGTTTACACCTCTAATGAGACCACAGATGT

rIBV-wt_P4_1764-1773 GTATATTTAAATGGTGATCTTGTTTACACCTCTAATGAGACCACAGATGT

mIBV_1B3IIA_P6_excl_S_ed --------------------------------------------------

20862

IBV_H52_BI TACATCTGCAGGTGTTTATTTTAAAGCTGGTGGACCTATAACTTATAAAG

rIBV-wt_P4_1764-1773 TACATCTGCAGGTGTTTATTTTAAAGCTGGTGGACCTATAACTTATAAAG

mIBV_1B3IIA_P6_excl_S_ed --------------------------------------------------

20912

IBV_H52_BI TTATGAGAGAAGTTAGAGCCCTGGCTTATTTTGTTAATGGTACTGCACAA

rIBV-wt_P4_1764-1773 TTATGAGAGAAGTTAGAGCCCTGGCTTATTTTGTTAATGGTACTGCACAA

mIBV_1B3IIA_P6_excl_S_ed --------------------------------------------------

20962

IBV_H52_BI GATGTTATTTTGTGTGATGGGTCACCTAGAGGCTTGTTAGCATGCCAGTA

rIBV-wt_P4_1764-1773 GATGTTATTTTGTGTGATGGGTCACCTAGAGGCTTGTTAGCATGCCAGTA

mIBV_1B3IIA_P6_excl_S_ed --------------------------------------------------

21012

IBV_H52_BI TAATACTGGCAATTTTTCAGATGGCTTTTATCCTTTTACTAATAGTAGTT

rIBV-wt_P4_1764-1773 TAATACTGGCAATTTTTCAGATGGCTTTTATCCTTTTACTAATAGTAGTT

mIBV_1B3IIA_P6_excl_S_ed --------------------------------------------------

21062

IBV_H52_BI TAGTTAAGCAGAAGTTTATTGTCTATCGTGAAAATAGTGTTAATACTACT

rIBV-wt_P4_1764-1773 TAGTTAAGCAGAAGTTTATTGTCTATCGTGAAAATAGTGTTAATACTACT

mIBV_1B3IIA_P6_excl_S_ed --------------------------------------------------

21112

IBV_H52_BI TTTACGTTACACAATTTCACTTTTCATAATGAGACTGGCGCCAACCCAAA

rIBV-wt_P4_1764-1773 TTTACGTTACACAATTTCACTTTTCATAATGAGACTGGCGCCAACCCAAA

mIBV_1B3IIA_P6_excl_S_ed --------------------------------------------------

21162

IBV_H52_BI TCCTAGTGGTGTCCAGAATATTCAAACTTACCAAACACAAACAGCTCAGA

rIBV-wt_P4_1764-1773 TCCTAGTGGTGTCCAGAATATTCAAACTTACCAAACACAAACAGCTCAGA

mIBV_1B3IIA_P6_excl_S_ed --------------------------------------------------

21212

IBV_H52_BI GTGGTTATTATAATTTTAATTTTTCCTTTCTGAGTAGTTTTGTTTATAAG

rIBV-wt_P4_1764-1773 GTGGTTATTATAATTTTAATTTTTCCTTTCTGAGTAGTTTTGTTTATAAG

mIBV_1B3IIA_P6_excl_S_ed --------------------------------------------------

21262

IBV_H52_BI GAGTCTAATTTTATGTATGGATCTTATCACCCAAGTTGTAATTTTAGACT

rIBV-wt_P4_1764-1773 GAGTCTAATTTTATGTATGGATCTTATCACCCAAGTTGTAATTTTAGACT

mIBV_1B3IIA_P6_excl_S_ed --------------------------------------------------

21312

IBV_H52_BI AGAAACTATTAATAATGGTTTGTGGTTTAATTCACTTTCAGTTTCAATTG

rIBV-wt_P4_1764-1773 AGAAACTATTAATAATGGTTTGTGGTTTAATTCACTTTCAGTTAGTATTG

mIBV_1B3IIA_P6_excl_S_ed --------------------------------------------------

21362

IBV_H52_BI CTTACGGTCCTCTTCAAGGTGGTTGCAAGCAATCTGTCTTTAGTGGTAGA

rIBV-wt_P4_1764-1773 CTTACGGTCCTCTTCAAGGTGGTTGCAAGCAATCTGTCTTTAGTGGTAGA

mIBV_1B3IIA_P6_excl_S_ed --------------------------------------------------

21412

IBV_H52_BI GCAACCTGTTGTTATGCTTACTCATATGGAGGTCCTTTGCTGTGTAAAGG

rIBV-wt_P4_1764-1773 GCAACCTGTTGTTATGCTTACTCATATGGAGGTCCTTTGCTGTGTAAAGG

mIBV_1B3IIA_P6_excl_S_ed --------------------------------------------------

21462

IBV_H52_BI TGTTTATTCAGGTGAGTTAGATCATAATTTTGAATGTGGACTGTTAGTTT

rIBV-wt_P4_1764-1773 TGTTTATTCAGGTGAGTTAGATCATAATTTTGAATGTGGACTGTTAGTTT

mIBV_1B3IIA_P6_excl_S_ed --------------------------------------------------

21512

IBV_H52_BI ATGTTACTAAGAGCGGTGGCTCTCGTATACAAACAGCCACTGAACCGCCA

rIBV-wt_P4_1764-1773 ATGTTACTAAGAGCGGTGGCTCTCGTATACAAACAGCCACTGAACCGCCA

mIBV_1B3IIA_P6_excl_S_ed --------------------------------------------------

21562

IBV_H52_BI GTTATAACTCAACACAATTATAATAATATTACTTTAAATACTTGTGTTGA

rIBV-wt_P4_1764-1773 GTTATAACTCAACACAATTATAATAATATTACTTTAAATACTTGTGTTGA

mIBV_1B3IIA_P6_excl_S_ed --------------------------------------------------

21612

IBV_H52_BI TTATAATATATATGGCAGAACTGGCCAAGGTTTTATTACTAATGTAACCG

rIBV-wt_P4_1764-1773 TTATAATATATATGGCAGAACTGGCCAGGGTTTTATTACTAATGTAACCG

mIBV_1B3IIA_P6_excl_S_ed --------------------------------------------------

21662

IBV_H52_BI ACTCAGCTGTTAGTTATAATTATCTAGCAGACGCAGGTTTGGCTATTTTA

rIBV-wt_P4_1764-1773 ACTCAGCTGTTAGTTATAATTATCTAGCAGACGCAGGTTTGGCTATTTTA

mIBV_1B3IIA_P6_excl_S_ed --------------------------------------------------

21712

IBV_H52_BI GATACATCTGGTTCCATAGACATCTTTGTCGTACAAAGTGAATATGGTCT

rIBV-wt_P4_1764-1773 GATACATCTGGTTCCATAGACATCTTTGTCGTACAAAGTGAATATGGTCT

mIBV_1B3IIA_P6_excl_S_ed --------------------------------------------------

21762

IBV_H52_BI TAATTATTATAAGGTTAACCCTTGCGAAGATGTCAACCAGCAGTTTGTAG

rIBV-wt_P4_1764-1773 TAATTATTATAAGGTTAACCCTTGCGAAGATGTCAACCAGCAGTTTGTAG

mIBV_1B3IIA_P6_excl_S_ed --------------------------------------------------

21812

IBV_H52_BI TTTCTGGTGGTAAATTAGTAGGTATTCTTACTTCACGTAATGAGACTGGT

rIBV-wt_P4_1764-1773 TTTCTGGTGGTAAATTAGTAGGTATTCTTACTTCACGTAATGAGACTGGT

mIBV_1B3IIA_P6_excl_S_ed --------------------------------------------------

21862

IBV_H52_BI TCCCAGCTTCTTGAGAATCAGTTTTACATCAAAATCACTAATGGAACACG

rIBV-wt_P4_1764-1773 TCCCAGCTTCTTGAGAATCAGTTTTACATCAAAATCACTAATGGAACACG

mIBV_1B3IIA_P6_excl_S_ed --------------------------------------------------

21912

IBV_H52_BI TCGTTTTAGACGTTCTATTACTGAAAGTGTTGAAAATTGCCCTTATGTTA

rIBV-wt_P4_1764-1773 TCGTTTTAGACGTTCTATTACTGAAAGTGTTGAAAATTGCCCTTATGTTA

mIBV_1B3IIA_P6_excl_S_ed --------------------------------------------------

21962

IBV_H52_BI GTTATGGTAAGTTTTGTATAAAACCTGATGGCTCAATTGCCACAATAGTA

rIBV-wt_P4_1764-1773 GTTATGGTAAGTTTTGTATAAAACCTGATGGCAGTATTGCCACAATAGTA

mIBV_1B3IIA_P6_excl_S_ed --------------------------------------------------

22012

IBV_H52_BI CCAAAACAATTGGAACAGTTTGTGGCACCTTTACTTAATGTTACTGAAAA

rIBV-wt_P4_1764-1773 CCAAAACAGTTAGAACAGTTTGTGGCACCTTTACTTAATGTTACTGAAAA

mIBV_1B3IIA_P6_excl_S_ed --------------------------------------------------

22062

IBV_H52_BI TGTGCTCATACCTAACAGTTTTAATTTAACTGTTACAGATGAGTACATAC

rIBV-wt_P4_1764-1773 TGTGCTCATACCTAACAGTTTTAATTTAACTGTTACAGATGAGTACATAC

mIBV_1B3IIA_P6_excl_S_ed --------------------------------------------------

22112

IBV_H52_BI AAACGCGTATGGATAAGGTCCAAATTAATTGCCTGCAGTATATTTGTGGC

rIBV-wt_P4_1764-1773 AAACTCGGATGGATAAGGTCCAAATTAATTGCCTGCAGTATATTTGTGGC

mIBV_1B3IIA_P6_excl_S_ed --------------------------------------------------

22162

IBV_H52_BI AATTCTCTGGAGTGCAGAAATTTGTTTCAACAATATGGTCCTGTTTGCGA

rIBV-wt_P4_1764-1773 AATTCTCTGGAGTGCAGAAATTTGTTTCAACAATATGGTCCTGTTTGCGA

mIBV_1B3IIA_P6_excl_S_ed --------------------------------------------------

22212

IBV_H52_BI CAACATATTGTCTGTAGTAAATAGTGTTGGTCAAAAAGAAGATATGGAAC

rIBV-wt_P4_1764-1773 CAACATATTGTCTGTAGTAAATAGTGTTGGTCAAAAAGAAGATATGGAAC

mIBV_1B3IIA_P6_excl_S_ed --------------------------------------------------

22262

IBV_H52_BI TTTTGAATTTCTATTCTTCTACTAAGCCGGCTGGTTTTAATACACCAGTT

rIBV-wt_P4_1764-1773 TTTTGAATTTCTATTCTTCTACTAAGCCGGCTGGTTTTAATACACCAGTT

mIBV_1B3IIA_P6_excl_S_ed --------------------------------------------------

22312

IBV_H52_BI CTTAGTAATGTTAGCACTGGTGAGTTTAATATTACTCTTTTTTTAACAAC

rIBV-wt_P4_1764-1773 CTTAGTAATGTTAGCACTGGTGAGTTTAATATTACTCTTTTTTTAACAAC

mIBV_1B3IIA_P6_excl_S_ed --------------------------------------------------

22362

IBV_H52_BI GCCTAGTAGTCCTAGAAGGCGTTCTTTTATTGAAGACCTTCTATTTACAA

rIBV-wt_P4_1764-1773 GCCTAGTAGTCCTAGAAGGCGTTCTTTTATTGAAGACCTTCTATTTACAA

mIBV_1B3IIA_P6_excl_S_ed --------------------------------------------------

22412

IBV_H52_BI GTGTTGAATCTGTTGGATTACCAACAGATGACGCATACAAAAATTGCACT

rIBV-wt_P4_1764-1773 GTGTTGAATCTGTTGGATTACCAACAGATGACGCATACAAAAATTGCACT

mIBV_1B3IIA_P6_excl_S_ed --------------------------------------------------

22462

IBV_H52_BI GCAGGTCCTTTAGGCTTTCTTAAGGACCTTGCATGTGCTCGTGAATATAA

rIBV-wt_P4_1764-1773 GCAGGTCCTTTAGGCTTTCTGAAAGACCTTGCATGTGCTCGTGAATATAA

mIBV_1B3IIA_P6_excl_S_ed --------------------------------------------------

22512

IBV_H52_BI TGGTTTGCTTGTGTTGCCTCCTATTATAACAGCAGAAATGCAAACTTTGT

rIBV-wt_P4_1764-1773 TGGTTTGCTTGTGTTGCCTCCTATTATAACAGCAGAAATGCAAACTTTGT

mIBV_1B3IIA_P6_excl_S_ed --------------------------------------------------

22562

IBV_H52_BI ATACTAGTTCTCTAGTAGCTTCTATGGCTTTTGGTGGTATTACTGCAGCT

rIBV-wt_P4_1764-1773 ATACAAGCTCTCTAGTAGCTTCTATGGCTTTTGGTGGTATTACTGCAGCT

mIBV_1B3IIA_P6_excl_S_ed --------------------------------------------------

22612

IBV_H52_BI GGTGCTATACCTTTTGCCACACAACTGCAGGCTAGAATTAATCACTTGGG

rIBV-wt_P4_1764-1773 GGTGCTATACCTTTTGCCACACAACTGCAGGCCAGAATTAATCACTTGGG

mIBV_1B3IIA_P6_excl_S_ed --------------------------------------------------

22662

IBV_H52_BI TATTACCCAGTCACTTCTTTTGAAGAATCAAGAAAAAATTGCTGCTTCCT

rIBV-wt_P4_1764-1773 TATTACCCAGTCACTTCTTTTGAAGAATCAAGAAAAAATTGCTGCTTCCT

mIBV_1B3IIA_P6_excl_S_ed --------------------------------------------------

22712

IBV_H52_BI TTAATAAGGCCATCGGTCATATGCAGGAAGGTTTTAGAAGTACATCTTTA

rIBV-wt_P4_1764-1773 TTAATAAGGCCATCGGTCATATGCAGGAAGGTTTTAGAAGTACATCTTTA

mIBV_1B3IIA_P6_excl_S_ed --------------------------------------------------

22762

IBV_H52_BI GCATTACAACAAATTCAAGATGTTGTTAATAAGCAGAGTGCTATTCTTAC

rIBV-wt_P4_1764-1773 GCATTACAACAAATTCAAGATGTTGTTAATAAGCAGAGTGCTATTCTTAC

mIBV_1B3IIA_P6_excl_S_ed --------------------------------------------------

22812

IBV_H52_BI TGAGACTATGGCATCACTTAATAAAAATTTTGGTGCCATTTCTTCTGTGA

rIBV-wt_P4_1764-1773 TGAGACTATGGCATCACTTAATAAAAATTTTGGTGCCATTTCTTCTGTGA

mIBV_1B3IIA_P6_excl_S_ed --------------------------------------------------

22862

IBV_H52_BI TTCAAGAAATCTACCAGCAACTTGACGCCATACAAGCAAATGCTCAAGTG

rIBV-wt_P4_1764-1773 TTCAAGAAATCTACCAGCAACTTGACGCCATACAAGCAAATGCTCAAGTG

mIBV_1B3IIA_P6_excl_S_ed --------------------------------------------------

22912

IBV_H52_BI GATCGTCTTATAACTGGTAGATTGTCATCACTTTCTGTTTTAGCATCTGC

rIBV-wt_P4_1764-1773 GATCGTCTTATAACTGGTAGATTGTCATCACTTTCTGTTTTAGCATCTGC

mIBV_1B3IIA_P6_excl_S_ed --------------------------------------------------

22962

IBV_H52_BI TAAGCAGGCGGAGTATATTAGAGTGTCACAACAGCGTGAGTTAGCTACTC

rIBV-wt_P4_1764-1773 TAAGCAGGCGGAGTATATTAGAGTGTCACAACAGCGTGAGTTAGCTACTC

mIBV_1B3IIA_P6_excl_S_ed --------------------------------------------------

23012

IBV_H52_BI AGAAGATTAATGAGTGTGTTAAGTCACAGTCCATTAGGTACTCCTTTTGT

rIBV-wt_P4_1764-1773 AGAAGATTAATGAGTGTGTTAAGTCACAGTCCATTAGGTACTCCTTTTGT

mIBV_1B3IIA_P6_excl_S_ed --------------------------------------------------

23062

IBV_H52_BI GGTAATGGACGACATGTTTTAACCATACCGCAAAATGCACCTAATGGTAT

rIBV-wt_P4_1764-1773 GGTAATGGACGACATGTTTTAACCATACCGCAAAATGCACCTAATGGTAT

mIBV_1B3IIA_P6_excl_S_ed --------------------------------------------------

23112

IBV_H52_BI AGTGTTTATACACTTTTCTTACACTCCAGATAGTTTTGTTAATGTTACTG

rIBV-wt_P4_1764-1773 AGTGTTTATACACTTTTCTTACACTCCAGATAGTTTTGTTAATGTTACTG

mIBV_1B3IIA_P6_excl_S_ed --------------------------------------------------

23162

IBV_H52_BI CAATAGTGGGTTTTTGTGTAAAGCCAGCTAATGCTAGTCAGTATGCAATA

rIBV-wt_P4_1764-1773 CAATAGTGGGTTTTTGTGTAAAGCCAGCTAATGCTAGTCAGTATGCAATA

mIBV_1B3IIA_P6_excl_S_ed --------------------------------------------------

23212

IBV_H52_BI GTACCCGCTAATGGTAGGGGTATTTTTATACAAGTTAATGGTAGTTACTA

rIBV-wt_P4_1764-1773 GTACCCGCTAATGGTAGGGGTATTTTTATACAAGTTAATGGTAGTTACTA

mIBV_1B3IIA_P6_excl_S_ed --------------------------------------------------

23262

IBV_H52_BI CATCACTGCACGAGATATGTATATGCCAAGAGCTATTACTGCAGGAGATA

rIBV-wt_P4_1764-1773 CATCACTGCACGAGATATGTATATGCCAAGAGCTATTACTGCAGGAGATA

mIBV_1B3IIA_P6_excl_S_ed --------------------------------------------------

23312

IBV_H52_BI TAGTTACGCTTACTTCTTGTCAAGCAAATTATGTAAGTGTAAATAAGACC

rIBV-wt_P4_1764-1773 TAGTTACGCTTACTTCTTGTCAAGCAAATTATGTAAGTGTAAATAAGACC

mIBV_1B3IIA_P6_excl_S_ed --------------------------------------------------

23362

IBV_H52_BI GTCATTACTACATTCGTAGACAATGATGATTTTGATTTTAATGACGAATT

rIBV-wt_P4_1764-1773 GTCATTACTACATTCGTAGACAATGATGATTTTGATTTTAATGACGAATT

mIBV_1B3IIA_P6_excl_S_ed --------------------------------------------------

23412

IBV_H52_BI GTCAAAATGGTGGAATGATACTAAGCATGAGCTACCAGACTTTGACAAAT

rIBV-wt_P4_1764-1773 GTCAAAATGGTGGAATGATACTAAGCATGAGCTACCAGACTTTGACAAAT

mIBV_1B3IIA_P6_excl_S_ed --------------------------------------------------

23462

IBV_H52_BI TCAATTACACAGTACCTATACTTGACATTGATAGTGAAATTGATCGTATT

rIBV-wt_P4_1764-1773 TCAATTACACAGTACCTATACTTGACATTGATAGTGAAATTGATCGTATT

mIBV_1B3IIA_P6_excl_S_ed --------------------------------------------------

23512

IBV_H52_BI CAAGGCGTTATACAGGGTCTTAATGACTCTCTAATAGACCTTGAAAAACT

rIBV-wt_P4_1764-1773 CAAGGCGTTATACAGGGTCTTAATGACTCTCTAATAGACCTTGAAAAACT

mIBV_1B3IIA_P6_excl_S_ed --------------------------------------------------

23562 -StyI- -NheI-

IBV_H52_BI TTCAATACTCAAAACTTATATTAAGTGGCCTTGGTATGTGTGGTTAGCCA

rIBV-wt_P4_1764-1773 TTCAATACTCAAAACTTATATTAAGTGGCCTTGGTATGTGTGGCTAGCCA

mIBV_1B3IIA_P6_excl_S_ed ----------------------------CCTTGGTATGTGTGGCTAGCCA

*************** ******

23612

IBV_H52_BI TAGCTTTTGCCACTATTATCTTCATCTTAATATTAGGATGGGTTTTCTTC

rIBV-wt_P4_1764-1773 TAGCTTTTGCCACTATTATCTTCATCTTAATATTAGGATGGGTTTTCTTC

mIBV_1B3IIA_P6_excl_S_ed TAGCTTTTGCCACTATTATCTTCATCTTAATATTAGGATGGGTTTTCTTC

**************************************************

23662

IBV_H52_BI ATGACTGGGTGTTGTGGTTGTTGTTGTGGATGCTTTGGCATTATGCCTCT

rIBV-wt_P4_1764-1773 ATGACTGGGTGTTGTGGTTGTTGTTGTGGATGCTTTGGCATTATGCCTCT

mIBV_1B3IIA_P6_excl_S_ed ATGACTGGGTGTTGTGGTTGTTGTTGTGGATGCTTTGGCATTATGCCTCT

**************************************************

23712

IBV_H52_BI AATGAGTAAGTGTGGTAAGAAATCTTCTTATTACACGACTTTTGATAACG

rIBV-wt_P4_1764-1773 AATGAGTAAGTGTGGTAAGAAATCTTCTTATTACACGACTTTTGATAACG

mIBV_1B3IIA_P6_excl_S_ed AATGAGTAAGTGTGGTAAGAAATCTTCTTATTACACGACTTTTGATAACG

**************************************************

23762

IBV_H52_BI ATGTGGTAACTGAACAATACAGACCTAAAAAGTCTGTTTAATGATCCAAA

rIBV-wt_P4_1764-1773 ATGTGGTAACTGAACAATACAGACCTAAAAAGTCTGTTTAATGATCCAAA

mIBV_1B3IIA_P6_excl_S_ed ATGTGGTAACTGAACAATACAGACCTAAAAAGTCTGTTTAATGATCCAAA

**************************************************

23812-SpeI-

IBV_H52_BI GTCCCACGTCCTTCTTAATAGTATTAATTTTGCTTTGGTGTAAACTTGTA

rIBV-wt_P4_1764-1773 GTCCCACTAGTTTCTTAATAGTATTAATTTTGCTTTGGTGTAAACTTGTA

mIBV_1B3IIA_P6_excl_S_ed GTCCCACTAGTTTCTTAATAGTATTAATTTTGCTTTGGTGTAAACTTGTA

******* ***************************************

23862 -AfeI-

IBV_H52_BI CTAAGTTGTTTTAGAGAGTTTATTATAGCGCTTCAACAACTAACACAAGT

rIBV-wt_P4_1764-1773 CTAAGTTGTTTTAGAGAGTTTATTATTGCCCTTCAACAACTAACACAAGT

mIBV_1B3IIA_P6_excl_S_ed CTAAGTTGTTTTAGAGAGTTTATTATTGCCCTTCAACAACTAACACAAGT

************************** ** ********************

23912

IBV_H52_BI TTTACTCCAAATTATCGATAGTAATTTACAGTCTAGACTGACCCTTTGGC

rIBV-wt_P4_1764-1773 TTTACTCCAAATTATCGATAGTAATTTACAGTCTAGACTGACCCTTTGGC

mIBV_1B3IIA_P6_excl_S_ed TTTACTCCAAATTATCGATAGTAATTTACAGTCTAGACTGACCCTTTGGC

**************************************************

23962 -AgeI-

IBV_H52_BI ACAGTCTAGACTAATGTTAAACTTAGAAGCAATTATTGAAACTGGTGATC

rIBV-wt_P4_1764-1773 ACAGTCTAGACTAATGTTAAACTTAGAAGCAATTATTGAAACCGGTGATC

mIBV_1B3IIA_P6_excl_S_ed ACAGTCTAGACTAATGTTAAACTTAGAAGCAATTATTGAAACCGGTGATC

****************************************** *******

24012

IBV_H52_BI AAGTGATTCAAAAAATCAGTTTCAATTTACAGCATATTTCAAGTGTATTA

rIBV-wt_P4_1764-1773 AAGTGATTCAAAAAATCAGTTTCAATTTACAGCATATTTCAAGTGTATTA

mIBV_1B3IIA_P6_excl_S_ed AAGTGATTCAAAAAATCAGTTTCAATTTACAGCATATTTCAAGTGTATTA

**************************************************

24062

IBV_H52_BI AACACAGAAGTATTTGACCCCTTTGACTATTGTTATTACAGAGGAGGTAA

rIBV-wt_P4_1764-1773 AACACAGAAGTATTTGACCCCTTTGACTATTGTTATTACAGAGGAGGTAA

mIBV_1B3IIA_P6_excl_S_ed AACACAGAAGTATTTGACCCCTTTGACTATTGTTATTACAGAGGAGGTAA

**************************************************

24112

IBV_H52_BI TTTTTGGGAAATAGAGTCAGCTGAAGATTGTTCAGGTGATGATGAATTTA

rIBV-wt_P4_1764-1773 TTTTTGGGAAATAGAGTCAGCTGAAGATTGTTCAGGTGATGATGAATTTA

mIBV_1B3IIA_P6_excl_S_ed TTTTTGGGAAATAGAGTCAGCTGAAGATTGTTCAGGTGATGATGAATTTA

**************************************************

24162 -AfeI-

IBV_H52_BI TTGAATAAGTCGCTAGAGGAGAATGGAAGTTTTCTAACAGCGCTTTACAT

rIBV-wt_P4_1764-1773 TTGAATAAGTCGCTAGAGGAGAATGGAAGTTTTCTAACGGCACTTTACAT

mIBV_1B3IIA_P6_excl_S_ed TTGAATAAGTCGCTAGAGGAGAATGGAAGTTTTCTAACGGCACTTTACAT

************************************** ** ********

24212

IBV_H52_BI ATTTGTAGGATTTTTAGCATTTTATCTTCTAGGTAGAGCACTTCAAGCAT

rIBV-wt_P4_1764-1773 ATTTGTAGGATTTTTAGCATTTTATCTTCTAGGTAGAGCACTTCAAGCAT

mIBV_1B3IIA_P6_excl_S_ed ATTTGTAGGATTTTTAGCATTTTATCTTCTAGGTAGAGCACTTCAAGCAT

**************************************************

24262 -PmlI-

IBV_H52_BI TTGTACAGGCTGCTGATGCTTGTTGTTTATTTTGGTATACATGGTTAGTA

rIBV-wt_P4_1764-1773 TTGTACAGGCTGCTGATGCTTGTTGTTTATTTTGGTACACGTGGTTAGTA

mIBV_1B3IIA_P6_excl_S_ed TTGTACAGGCTGCTGATGCTTGTTGTTTATTTTGGTACACGTGGTTAGTA

************************************* ** *********

24312

IBV_H52_BI ATTCCAGGAGTTAAGGGTACAGCCTTTGTATACAAGTATACATATGGTAG

rIBV-wt_P4_1764-1773 ATTCCAGGAGTTAAGGGTACAGCCTTTGTATACAAGTATACATATGGTAG

mIBV_1B3IIA_P6_excl_S_ed ATTCCAGGAGTTAAGGGTACAGCCTTTGTATACAAGTATACATATGGTAG

**************************************************

24362

IBV_H52_BI AAAACTTAACAATTCGGAATTAGAAGCAGTTGTTGTTAACGAGTTTCCTA

rIBV-wt_P4_1764-1773 AAAACTTAACAATTCGGAATTAGAAGCAGTTGTTGTTAACGAGTTTCCTA

mIBV_1B3IIA_P6_excl_S_ed AAAACTTAACAATTCGGAATTAGAAGCAGTTGTTGTTAACGAGTTTCCTA

**************************************************

24412

IBV_H52_BI AGAACGGTTGGAATAATAAAAATCCAGCAAATTTTCAAGATGTCCAACGA

rIBV-wt_P4_1764-1773 AGAACGGTTGGAATAATAAAAATCCAGCAAATTTTCAAGATGTCCAACGA

mIBV_1B3IIA_P6_excl_S_ed AGAACGGTTGGAATAATAAAAATCCAGCAAATTTTCAAGATGTCCAACGA

**************************************************

24462

IBV_H52_BI AACAAATTGTACTCTTGACTTTGAACAGTCAGTTGAGCTTTTTAAAGAGT

rIBV-wt_P4_1764-1773 AACAAATTGTACTCTTGACTTTGAACAGTCAGTTGAGCTTTTTAAAGAGT

mIBV_1B3IIA_P6_excl_S_ed AACAAATTGTACTCTTGACTTTGAACAGTCAGTTGAGCTTTTTAAAGAGT

**************************************************

24512

IBV_H52_BI ATAATTTATTTATAACTGCATTCTTGTTGTTCTTAACCATAATACTTCAG

rIBV-wt_P4_1764-1773 ATAATTTATTTATAACTGCATTCTTGTTGTTCTTAACCATAATACTTCAG

mIBV_1B3IIA_P6_excl_S_ed ATAATTTATTTATAACTGCATTCTTGTTGTTCTTAACCATAATACTTCAG

**************************************************

24562 -MluI-

IBV_H52_BI TATGGTTATGCAACAAGAAGTAAGTTTATTTATATACTTAAAATGATAGT

rIBV-wt_P4_1764-1773 TATGGTTATGCAACGCGTAGTAAGTTTATTTATATACTTAAAATGATAGT

mIBV_1B3IIA_P6_excl_S_ed TATGGTTATGCAACGCGTAGTAAGTTTATTTATATACTTAAAATGATAGT

************** * ********************************

24612

IBV_H52_BI GTTATGGTGCTTTTGGCCCCTTAACATTGCAGTAGGTGTAATTTCATGTA

rIBV-wt_P4_1764-1773 GTTATGGTGCTTTTGGCCCCTTAACATTGCAGTAGGTGTAATTTCATGTA

mIBV_1B3IIA_P6_excl_S_ed GTTATGGTGCTTTTGGCCCCTTAACATTGCAGTAGGTGTAATTTCATGTA

**************************************************

24662

IBV_H52_BI TATACCCACCAAACACAGGAGGTCTTGTCGCAGCGATAATACTTACTGTG

rIBV-wt_P4_1764-1773 TATACCCACCAAACACAGGAGGTCTTGTCGCAGCGATAATACTTACTGTG

mIBV_1B3IIA_P6_excl_S_ed TATACCCACCAAACACAGGAGGTCTTGTCGCAGCGATAATACTTACTGTG

**************************************************

24712

IBV_H52_BI TTTGCGTGTCTTTCTTTTGTAGGTTATTGGATCCAGAGTATTAGACTCTT

rIBV-wt_P4_1764-1773 TTTGCGTGTCTTTCTTTTGTAGGTTATTGGATCCAGAGTATTAGACTCTT

mIBV_1B3IIA_P6_excl_S_ed TTTGCGTGTCTTTCTTTTGTAGGTTATTGGATCCAGAGTATTAGACTCTT

**************************************************

24762

IBV_H52_BI TAAGCGGTGTAGATCTTGGTGGTCATTTAACCCAGAATCTAACGCCGTAG

rIBV-wt_P4_1764-1773 TAAGCGGTGTAGATCTTGGTGGTCATTTAACCCAGAATCTAACGCCGTAG

mIBV_1B3IIA_P6_excl_S_ed TAAGCGGTGTAGATCTTGGTGGTCATTTAACCCAGAATCTAACGCCGTAG

**************************************************

24812

IBV_H52_BI GTTCAATACTCCTAACTAATGGTCAACAATGTAATTTTGCTATAGAGAGT

rIBV-wt_P4_1764-1773 GTTCAATACTCCTAACTAATGGTCAACAATGTAATTTTGCTATAGAGAGT

mIBV_1B3IIA_P6_excl_S_ed GTTCAATACTCCTAACTAATGGTCAACAATGTAATTTTGCTATAGAGAGT

**************************************************

24862

IBV_H52_BI GTGCCGATGGTGCTTTCTCCTATTATAAAGAATGGTGTTCTTTATTGTGA

rIBV-wt_P4_1764-1773 GTGCCGATGGTGCTTTCTCCTATTATAAAGAATGGTGTTCTTTATTGTGA

mIBV_1B3IIA_P6_excl_S_ed GTGCCGATGGTGCTTTCTCCTATTATAAAGAATGGTGTTCTTTATTGTGA

**************************************************

24912

IBV_H52_BI GGGTCAGTGGCTTGCTAAATGTGAACCAGACCACTTGCCTAAAGACATAT

rIBV-wt_P4_1764-1773 GGGTCAGTGGCTTGCTAAATGTGAACCAGACCACTTGCCTAAAGACATAT

mIBV_1B3IIA_P6_excl_S_ed GGGTCAGTGGCTTGCTAAATGTGAACCAGACCACTTGCCTAAAGACATAT

**************************************************

24962

IBV_H52_BI TTGTATGCACACCAGATAGACGTAATATCTATCGTATGGTGCAGAAATAC

rIBV-wt_P4_1764-1773 TTGTATGCACACCAGATAGACGTAATATCTATCGTATGGTGCAGAAATAC

mIBV_1B3IIA_P6_excl_S_ed TTGTATGCACACCAGATAGACGTAATATCTATCGTATGGTGCAGAAATAC

**************************************************

25012

IBV_H52_BI ACTGGTGACCAAAGCGGAAATAAGAAAAGGTTTGCTACATTTGTCTATGC

rIBV-wt_P4_1764-1773 ACTGGTGACCAAAGCGGAAATAAGAAAAGGTTTGCTACATTTGTCTATGC

mIBV_1B3IIA_P6_excl_S_ed ACTGGTGACCAAAGCGGAAATAAGAAAAGGTTTGCTACATTTGTCTATGC

**************************************************

25062

IBV_H52_BI AAAGCAGTCAGTAGACACTGGCGAGCTAGAAAGTGTAGCAACAGGTGGAA

rIBV-wt_P4_1764-1773 AAAGCAGTCAGTAGACACTGGCGAGCTAGAAAGTGTAGCAACAGGTGGAA

mIBV_1B3IIA_P6_excl_S_ed AAAGCAGTCAGTAGACACTGGCGAGCTAGAAAGTGTAGCAACAGGTGGAA

**************************************************

25112

IBV_H52_BI GTAGCCTTTACACATAAATGTGTGTGTGTAGAGAGTATTTAAAATTATTC

rIBV-wt_P4_1764-1773 GTAGCCTTTACACATAAATGTGTGTGTGTAGAGAGTATTTAAAATTATTC

mIBV_1B3IIA_P6_excl_S_ed GTAGCCTTTACACATAAATGTGTGTGTGTAGAGAGTATTTAAAATTATTC

**************************************************

25162

IBV_H52_BI TTCAATAGTGCCTCTATTTTAAGAGCGCGGAAGAGTATTTGTTTTGAGGA

rIBV-wt_P4_1764-1773 TTCAATAGTGCCTCTATTTTAAGAGCGCGGAAGAGTATTTGTTTTGAGGA

mIBV_1B3IIA_P6_excl_S_ed TTCAATAGTGCCTCTATTTTAAGAGCGCGGAAGAGTATTTGTTTTGAGGA

**************************************************

25212

IBV_H52_BI TATTAATATAAATCCTCTTTGTTTTGTACTCTCTTTACAAGAGTTATTAT

rIBV-wt_P4_1764-1773 TATTAATATAAATCCTCTTTGTTTTGTACTCTCTTTACAAGAGTTATTAT

mIBV_1B3IIA_P6_excl_S_ed TATTAATATAAATCCTCTTTGTTTTGTACTCTCTTTACAAGAGTTATTAT

**************************************************

25262

IBV_H52_BI TTAAGCAACAGTTTTTCCTTTCCTTTGTTTGGAAGAAAGTTGTTGTTAAT

rIBV-wt_P4_1764-1773 TTAAGCAACAGTTTTTCCTTTCCTTTGTTTGGAAGAAAGTTGTTGTTAAT

mIBV_1B3IIA_P6_excl_S_ed TTAAGCAACAGTTTTTCCTTTCCTTTGTTTGGAAGAAAGTTGTTGTTAAT

**************************************************

25312 EcoRI-

IBV_H52_BI GGTGTAGAATTCCAAGTAGAAAATGGAAAAGTCCACTACGAAGGAAACCC

rIBV-wt_P4_1764-1773 GGTGTAGAATTCCAAGTAGAAAATGGAAAAGTCCACTACGAAGGAAACCC

mIBV_1B3IIA_P6_excl_S_ed GGTGTAGAATTCCAAGTAGAAAATGGAAAAGTCCACTACGAAGGAAACCC

**************************************************

25362

IBV_H52_BI CATTTTCCAAAAAGGTTGTTGTAGGTTGTGGTCCCATTATAAGAAGGATT

rIBV-wt_P4_1764-1773 CATTTTCCAAAAAGGTTGTTGTAGGTTGTGGTCCCATTATAAGAAGGATT

mIBV_1B3IIA_P6_excl_S_ed CATTTTCCAAAAAGGTTGTTGTAGGTTGTGGTCCCATTATAAGAAGGATT

**************************************************

25412

IBV_H52_BI AAATGGATTAAACCACCTACACTACTTACTTGTAATAAGGGCGTTTGGAC

rIBV-wt_P4_1764-1773 AAATGGATTAAACCACCTACACTACTTACTTGTAATAAGGGCGTTTGGAC

mIBV_1B3IIA_P6_excl_S_ed AAATGGATTAAACCACCTACACTACTTACTTGTAATAAGGGCGTTTGGAC

**************************************************

25462-AfeI- -SpeI-

IBV_H52_BI TTACAAGCGCTTAACAAATACAGACGATGAAATGGCTGACTAGTTTTGGA

rIBV-wt_P4_1764-1773 TTACAAGCGCTTAACAAATACAGACGATGAAATGGCTGACTAGTTTTGGA

mIBV_1B3IIA_P6_excl_S_ed TTACAAGCGCTTAACAAATACAGACGATGAAATGGCTGACTAGTTTTGGA

**************************************************

25512

IBV_H52_BI AGAGCAGTTATTTCTTGTTATAAAGCCCTACTATTAACTCAGTTAAGAGT

rIBV-wt_P4_1764-1773 AGAGCAGTTATTTCTTGTTATAAAGCCCTACTATTAACTCAGTTAAGAGT

mIBV_1B3IIA_P6_excl_S_ed AGAGCAGTTATTTCTTGTTATAAAGCCCTACTATTAACTCAGTTAAGAGT

**************************************************

25562 -MluI-

IBV_H52_BI ATTAGATAGGTTAATTTTAGATCACGGACCAAAACGCGTCTTAACGTGTG

rIBV-wt_P4_1764-1773 ATTAGATAGGTTAATTTTAGATCACGGACCAAAGCGAGTCTTAACGTGTG

mIBV_1B3IIA_P6_excl_S_ed ATTAGATAGGTTAATTTTAGATCACGGACCAAAGCGAGTCTTAACGTGTG

********************************* ** *************

25612

IBV_H52_BI GTAGGCGAGTGCTTTTATCTCAATTAGATTTAGTTTATAGGTTGGCATAT

rIBV-wt_P4_1764-1773 GTAGGCGAGTGCTTTTATCTCAATTAGATTTAGTTTATAGGTTGGCATAT

mIBV_1B3IIA_P6_excl_S_ed GTAGGCGAGTGCTTTTATCTCAATTAGATTTAGTTTATAGGTTGGCATAT

**************************************************

25662

IBV_H52_BI ACGCCCACCCAATCGCTGGTATGAATAATAGTAAAGATAATCCTTTTCGC

rIBV-wt_P4_1764-1773 ACGCCCACCCAATCGCTGGTATGAATAATAGTAAAGATAATCCTTTTCGC

mIBV_1B3IIA_P6_excl_S_ed ACGCCCACCCAATCGCTGGTATGAATAATAGTAAAGATAATCCTTTTCGC

**************************************************

25712

IBV_H52_BI GGAGCAATAGCAAGAAAAGCGCGAATTTATCTGAGAGAAGGATTAGAGTG

rIBV-wt_P4_1764-1773 GGAGCAATAGCAAGAAAAGCGCGAATTTATCTGAGAGAAGGATTAGAGTG

mIBV_1B3IIA_P6_excl_S_ed GGAGCAATAGCAAGAAAAGCGCGAATTTATCTGAGAGAAGGATTAGAGTG

**************************************************

25762

IBV_H52_BI TGTTTACTTTCTTAACAAAGCAGGACAAGCAGAGCCTTGTCCCGCGTGTA

rIBV-wt_P4_1764-1773 TGTTTACTTTCTTAACAAAGCAGGACAAGCAGAGCCTTGTCCCGCGTGTA

mIBV_1B3IIA_P6_excl_S_ed TGTTTACTTTCTTAACAAAGCAGGACAAGCAGAGCCTTGTCCCGCGTGTA

**************************************************

25812 -StyI-

IBV_H52_BI CCTCCCTAGTATTCCAAGGGAAAACTTGTGAGGAACACACAGATAATAAT

rIBV-wt_P4_1764-1773 CCTCCCTAGTATTTCAGGGGAAAACTTGTGAGGAACACACAGATAATAAT

mIBV_1B3IIA_P6_excl_S_ed CCTCCCTAGTATTTCAGGGGAAAACTTGTGAGGAACACACAGATAATAAT

************* ** *********************************

25862

IBV_H52_BI AATCTTTTGTCATGGCGAGCGGTAAGACAACTGGGAAGACAGACGCCCCA

rIBV-wt_P4_1764-1773 AATCTTTTGTCATGGCGAGCGGTAAGACAACTGGGAAGACAGACGCCCCA

mIBV_1B3IIA_P6_excl_S_ed AATCTTTTGTCATGGCGAGCGGTAAGACAACTGGGAAGACAGACGCCCCA

**************************************************

25912

IBV_H52_BI GCGCCAGTCATCAAACTAGGAGGGCCAAAACCACCTAAAGTTGGTTCTTC

rIBV-wt_P4_1764-1773 GCGCCAGTCATCAAACTAGGAGGGCCAAAACCACCTAAAGTTGGTTCTTC

mIBV_1B3IIA_P6_excl_S_ed GCGCCAGTCATCAAACTAGGAGGGCCAAAACCACCTAAAGTTGGTTCTTC

**************************************************

25962 -NheI-

IBV_H52_BI TGGAAATGCATCTTGGTTTCAAGCACTAAAAGCCAAGAAGTTAAATTCAC

rIBV-wt_P4_1764-1773 TGGAAATGCTAGCTGGTTTCAAGCACTAAAAGCCAAGAAGTTAAATTCAC

mIBV_1B3IIA_P6_excl_S_ed TGGAAATGCTAGCTGGTTTCAAGCACTAAAAGCCAAGAAGTTAAATTCAC

********* *************************************

26012

IBV_H52_BI CTCCTCCTAAGTTTGAAGGTAGCGGCGTTCCTGATAATGAAAATCTTAAA

rIBV-wt_P4_1764-1773 CTCCTCCTAAGTTTGAAGGTAGCGGCGTTCCTGATAATGAAAATCTTAAA

mIBV_1B3IIA_P6_excl_S_ed CTCCTCCTAAGTTTGAAGGTAGCGGCGTTCCTGATAATGAAAATCTTAAA

**************************************************

26062

IBV_H52_BI TTAAGCCAGCAACATGGGTACTGGAGACGTCAAGCCAGGTACAAGCCAGG

rIBV-wt_P4_1764-1773 TTAAGCCAGCAACATGGGTACTGGAGACGTCAAGCCAGGTACAAGCCAGG

mIBV_1B3IIA_P6_excl_S_ed TTAAGCCAGCAACATGGGTACTGGAGACGTCAAGCCAGGTACAAGCCAGG

**************************************************

26112

IBV_H52_BI TAAAGGCGGAAGAAAATCAGTCCCAGATGCTTGGTACTTCTATTACACTG

rIBV-wt_P4_1764-1773 TAAAGGCGGAAGAAAATCAGTCCCAGATGCTTGGTACTTCTATTACACTG

mIBV_1B3IIA_P6_excl_S_ed TAAAGGCGGAAGAAAATCAGTCCCAGATGCTTGGTACTTCTATTACACTG

**************************************************

26162

IBV_H52_BI GAACAGGACCAGCCGCTGACCTGAATTGGGGTGATAGCCAAGATGGTATA

rIBV-wt_P4_1764-1773 GAACAGGACCAGCCGCTGACCTGAATTGGGGTGATAGCCAAGATGGTATA

mIBV_1B3IIA_P6_excl_S_ed GAACAGGACCAGCCGCTGACCTGAATTGGGGTGATAGCCAAGATGGTATA

**************************************************

26212

IBV_H52_BI GTGTGGGTTTCTGCAAAGGGTGCTGATACTAAATCTAGATCTAACCAGGG

rIBV-wt_P4_1764-1773 GTGTGGGTTTCTGCAAAGGGTGCTGATACTAAATCTAGATCTAACCAGGG

mIBV_1B3IIA_P6_excl_S_ed GTGTGGGTTTCTGCAAAGGGTGCTGATACTAAATCTAGATCTAACCAGGG

**************************************************

26262

IBV_H52_BI TACAAGGGATCCTGATAAGTTTGACCAATACCCGCTACGATTCTCAGATG

rIBV-wt_P4_1764-1773 TACAAGGGATCCTGATAAGTTTGACCAATACCCGCTACGATTCTCAGATG

mIBV_1B3IIA_P6_excl_S_ed TACAAGGGATCCTGATAAGTTTGACCAATACCCGCTACGATTCTCAGATG

**************************************************

26312

IBV_H52_BI GAGGACCTGATGGTAATTTCCGTTGGGACTTCATTCCAATAAATCGTGGT

rIBV-wt_P4_1764-1773 GAGGACCTGATGGTAATTTCCGTTGGGACTTCATTCCAATAAATCGTGGT

mIBV_1B3IIA_P6_excl_S_ed GAGGACCTGATGGTAATTTCCGTTGGGACTTCATTCCAATAAATCGTGGT

**************************************************

26362

IBV_H52_BI AGGAGTGGAAGATCAACAGCGGCTTCATCAGCAGCATCTAGTAGAGCACC

rIBV-wt_P4_1764-1773 AGGAGTGGAAGATCAACAGCGGCTTCATCAGCAGCATCTAGTAGAGCACC

mIBV_1B3IIA_P6_excl_S_ed AGGAGTGGAAGATCAACAGCGGCTTCATCAGCAGCATCTAGTAGAGCACC

**************************************************

26412

IBV_H52_BI GTCGCGTGATGGCTCGCGTGGACGTAGAAGCGGAGCTGAAGATGATCTTA

rIBV-wt_P4_1764-1773 GTCGCGTGATGGCTCGCGTGGACGTAGAAGCGGAGCTGAAGATGATCTTA

mIBV_1B3IIA_P6_excl_S_ed GTCGCGTGATGGCTCGCGTGGACGTAGAAGCGGAGCTGAAGATGATCTTA

**************************************************

26462

IBV_H52_BI TAGCTCGTGCAGCAAAGATCATTCAGGATCAGCAGAAGAAGGGTTCTCGC

rIBV-wt_P4_1764-1773 TAGCTCGTGCAGCAAAGATCATTCAGGATCAGCAGAAGAAGGGTTCTCGC

mIBV_1B3IIA_P6_excl_S_ed TAGCTCGTGCAGCAAAGATCATTCAGGATCAGCAGAAGAAGGGTTCTCGC

**************************************************

26512

IBV_H52_BI ATTACTAAAGCTAAGGCCGATGAAATGGCTCATCGCCGGTATTGTAAGCG

rIBV-wt_P4_1764-1773 ATTACTAAAGCTAAGGCCGATGAAATGGCTCATCGCCGGTATTGTAAGCG

mIBV_1B3IIA_P6_excl_S_ed ATTACTAAAGCTAAGGCCGATGAAATGGCTCATCGCCGGTATTGTAAGCG

**************************************************

26562

IBV_H52_BI TACTATCCCACCTGGTTATAAGGTTGATCAAGTATTTGGTCCCCGTACTA

rIBV-wt_P4_1764-1773 TACTATCCCACCTGGTTATAAGGTTGATCAAGTATTTGGTCCCCGTACTA

mIBV_1B3IIA_P6_excl_S_ed TACTATCCCACCTGGTTATAAGGTTGATCAAGTATTTGGTCCCCGTACTA

**************************************************

26612

IBV_H52_BI AAGGTAAGGAGGGAAATTTTGGTGATGACAAGATGAATGAGGAGGGTATT

rIBV-wt_P4_1764-1773 AAGGTAAGGAGGGAAATTTTGGTGATGACAAGATGAATGAGGAGGGTATT

mIBV_1B3IIA_P6_excl_S_ed AAGGTAAGGAGGGAAATTTTGGTGATGACAAGATGAATGAGGAGGGTATT

**************************************************

26662

IBV_H52_BI AAGGATGGGCGCGTTACAGCAATGCTCAACCTAGTCCCTAGCAGCCATGC

rIBV-wt_P4_1764-1773 AAGGATGGGCGCGTTACAGCAATGCTCAACCTAGTCCCTAGCAGCCATGC

mIBV_1B3IIA_P6_excl_S_ed AAGGATGGGCGCGTTACAGCAATGCTCAACCTAGTCCCTAGCAGCCATGC

**************************************************

26712

IBV_H52_BI TTGTCTTTTTGGAAGTAGAGTGACGCCCAAACTTCAACCAGATGGGCTGC

rIBV-wt_P4_1764-1773 TTGTCTTTTTGGAAGTAGAGTGACGCCCAAACTTCAACCAGATGGGCTGC

mIBV_1B3IIA_P6_excl_S_ed TTGTCTTTTTGGAAGTAGAGTGACGCCCAAACTTCAACCAGATGGGCTGC

**************************************************

26762 -PmlI-

IBV_H52_BI ACTTGAGATTTGAATTTACTACTGTGGTTTCACGTGATGATCCGCAGTTT

rIBV-wt_P4_1764-1773 ACTTGAGATTTGAATTTACTACTGTGGTTTCTAGGGATGATCCGCAGTTT

mIBV_1B3IIA_P6_excl_S_ed ACTTGAGATTTGAATTTACTACTGTGGTTTCTAGGGATGATCCGCAGTTT

******************************* * ***************

26812 -MluI

IBV_H52_BI GATAATTATGTGAAAATTTGTGATCAGTGTGTCGATGGTGTAGGGACGCG

rIBV-wt_P4_1764-1773 GATAATTATGTGAAAATTTGTGATCAGTGTGTCGATGGTGTAGGGACTCG

mIBV_1B3IIA_P6_excl_S_ed GATAATTATGTGAAAATTTGTGATCAGTGTGTCGATGGTGTAGGGACTCG

*********************************************** **

-

IBV_H52_BI TCCAAAAGACGATGAACCGAGACCAAAGTCACGCCCAAATTCAAGACCTG

rIBV-wt_P4_1764-1773 GCCAAAAGACGATGAACCGAGACCAAAGTCACGCCCAAATTCAAGACCTG

mIBV_1B3IIA_P6_excl_S_ed GCCAAAAGACGATGAACCGAGACCAAAGTCACGCCCAAATTCAAGACCTG

*************************************************

26912

IBV_H52_BI CTACAAGAACAAGTTCTCCAGCGCCAAGACAACAGCGTCAAAAGAAGGAG

rIBV-wt_P4_1764-1773 CTACAAGAACAAGTTCTCCAGCGCCAAGACAACAGCGTCAAAAGAAGGAG

mIBV_1B3IIA_P6_excl_S_ed CTACAAGAACAAGTTCTCCAGCGCCAAGACAACAGCGTCAAAAGAAGGAG

**************************************************

26962

IBV_H52_BI AAGAAGTCAAAGAAGCAGGATGATGAAGTAGATAAGGCATTGACCTCAGA

rIBV-wt_P4_1764-1773 AAGAAGTCAAAGAAGCAGGATGATGAAGTAGATAAGGCATTGACCTCAGA

mIBV_1B3IIA_P6_excl_S_ed AAGAAGTCAAAGAAGCAGGATGATGAAGTAGATAAGGCATTGACCTCAGA

**************************************************

27012 -StyI-

IBV_H52_BI TGAGGAGAGGAACAATGCACAGCTGGAATTTGATGATGAACCCAAGGTGA

rIBV-wt_P4_1764-1773 TGAGGAGAGGAACAATGCACAGCTGGAATTTGATGATGAACCGAAAGTGA

mIBV_1B3IIA_P6_excl_S_ed TGAGGAGAGGAACAATGCACAGCTGGAATTTGATGATGAACCGAAAGTGA

****************************************** ** ****

27062

IBV_H52_BI TTAACTGGGGGGATTCAGCACTTGGAGAGAATGAGTTGTAAAGCTAGATT

rIBV-wt_P4_1764-1773 TTAACTGGGGGGATTCAGCACTTGGAGAGAATGAGTTGTAAAGCTAGATT

mIBV_1B3IIA_P6_excl_S_ed TTAACTGGGGGGATTCAGCACTTGGAGAGAATGAGTTGTAAAGCTAGATT

**************************************************

27112

IBV_H52_BI TCCAACTTAACATCATGGACGTGCGTATGCTGTTTTTCCCTACTATAGAC

rIBV-wt_P4_1764-1773 TCCAACTTAACATCATGGACGTGCGTATGCTGTTTTTCCCTACTATAGAC

mIBV_1B3IIA_P6_excl_S_ed TCCAACTTAACATCATGGACGTGCGTATGCTGTTTTTCCCTACTATAGAC

**************************************************

27162

IBV_H52_BI TTTTTAGCATATTATTTTTTGCTATTTGTATGGTTTATTACAGGTGAAGA

rIBV-wt_P4_1764-1773 TTTTTAGCATATTATTTTTTGCTATTTGTATGGTTTATTACAGGTGAAGA

mIBV_1B3IIA_P6_excl_S_ed TTTTTAGCATATTATTTTTTGCTATTTGTATGGTTTATTACAGGTGAAGA

**************************************************

27212

IBV_H52_BI TTGTATGTATTTGTTGTACACTCGTATGTTCTATATTATGTTTTCTGTAG

rIBV-wt_P4_1764-1773 TTGTATGTATTTGTTGTACACTCGTATGTTCTATATTATGTTTTCTGTAG

mIBV_1B3IIA_P6_excl_S_ed TTGTATGTATTTGTTGTACACTCGTATGTTCTATATTATGTTTTCTGTAG

**************************************************

27262

IBV_H52_BI TTGTTATTAGTGTTGTTCTTGTTCTTACTCTACTGTTCTCTTTTCTTTAT

rIBV-wt_P4_1764-1773 TTGTTATTAGTGTTGTTCTTGTTCTTACTCTACTGTTCTCTTTTCTTTAT

mIBV_1B3IIA_P6_excl_S_ed TTGTTATTAGTGTTGTTCTTGTTCTTACTCTACTGTTCTCTTTTCTTTAT

**************************************************

27312

IBV_H52_BI TTTAGAGTATCAATAAGAATCAAGGAAGATAGGCATGTAGTTTGATTACC

rIBV-wt_P4_1764-1773 TTTAGAGTATCAATAAGAATCAAGGAAGATAGGCATGTAGTTTGATTACC

mIBV_1B3IIA_P6_excl_S_ed TTTAGAGTATCAATAAGAATCAAGGAAGATAGGCATGTAGTTTGATTACC

**************************************************

27362

IBV_H52_BI TACATGTCTATCGCCAGGGAAATGTCTAATCTGTCTACTTAGTAGCCTGG

rIBV-wt_P4_1764-1773 TACATGTCTATCGCCAGGGAAATGTCTAATCTGTCTACTTAGTAGCCTGG

mIBV_1B3IIA_P6_excl_S_ed TACATGTCTATCGCCAGGGAAATGTCTAATCTGTCTACTTAGTAGCCTGG

**************************************************

27412

IBV_H52_BI AAACGAACGGTAGACCCTTAGATTTTAATTTAGTTTAATTTTTAGTTTAG

rIBV-wt_P4_1764-1773 AAACGAACGGTAGACCCTTAGATTTTAATTTAGTTTAATTTTTAGTTTAG

mIBV_1B3IIA_P6_excl_S_ed AAACGAACGGTAGACCCTTAGATTTTAATTTAGTTTAATTTTTAGTTTAG

**************************************************

27462

IBV_H52_BI TTTAAGTTAGTTTAGAGTAGGTATAAAGAAGCCAGTGCCGGGGCCACGCG

rIBV-wt_P4_1764-1773 TTTAAGTTAGTTTAGAGTAGGTATAAAGAAGCCAGTGCCGGGGCCACGCG

mIBV_1B3IIA_P6_excl_S_ed TTTAAGTTAGTTTAGAGTAGGTATAAAGAAGCCAGTGCCGGGGCCACGCG

**************************************************

27512

IBV_H52_BI GAGTACGATCGAGGGTACAGCACTAGGACGCCCACTAGGGGAAGAGCTAA

rIBV-wt_P4_1764-1773 GAGTACGATCGAGGGTACAGCACTAGGACGCCCACTAGGGGAAGAGCTAA

mIBV_1B3IIA_P6_excl_S_ed GAGTACGATCGAGGGTACAGCACTAGGACGCCCACTAGGGGAAGAGCTAA

**************************************************

27562

IBV_H52_BI ATTTTAGTTTAAGTTAAGTTTAATTGGCTAAGTATAGTTAAAATTTATAG

rIBV-wt_P4_1764-1773 ATTTTAGTTTAAGTTAAGTTTAATTGGCTAAGTATAGTTAAAATTTATAG

mIBV_1B3IIA_P6_excl_S_ed ATTTTAGTTTAAGTTAAGTTTAATTGGCTAAGTATAGTTAAAATTTATAG

**************************************************

27612

IBV_H52_BI GCTAGTATAGAGTTAGAGCAAAAAAAAAA

rIBV-wt_P4_1764-1773 GCTAGTATAGAGTTAGAGCAAAAAAAAAA

mIBV_1B3IIA_P6_excl_S_ed GCTAGTATAGAGTTAGAGCAAAAAAAAAA

*****************************
